# A novel multidimensional strategy to evaluate *Belamcanda chinensis* (L) DC and *Iris tectorum* Maxim based on plant metabolomics, digital reference standard analyzer and biological activities evaluation

**DOI:** 10.1186/s13020-021-00494-3

**Published:** 2021-08-26

**Authors:** Hongxu Zhou, Yi Zhang, Hui Liang, Huijie Song, Jiaming Zhao, Li Liu, Jun Zeng, Lei Sun, Shuangcheng Ma, Dali Meng

**Affiliations:** 1 School of Traditional Chinese Materia Medica, Shenyang Pharmaceutical University, Shenyang, 110016 People’s Republic of China; 2 Chongqing Institute for Food and Drug Control, Chongqing, 401121 People’s Republic of China; 3 National Institutes for Food and Drug Control, Beijing, 100050 People’s Republic of China

**Keywords:** *Belamcanda chinensis* (L.) DC, *Iris tectorum* Maxim, Plant metabolomics, Digital reference standard, Biological activities

## Abstract

**Background:**

*Belamcanda chinensis* (L.) DC. (BC) belongs to the family of *Iridaceae* and is widely cultivated and used in many Chinese patent medicine and Chinese medicinal formulae. However, due to the high similarities in appearance such as color and shape to *Iris tectorum* Maxim (ITM), another plant from the same family, BC is often confused or even misused with ITM.

**Methods:**

Therefore, in order to distinguish the chemical constituents, qualities and biological activities of BC and ITM, multiple technologies including plant metabolomics, digital reference standard (DRS) analyzer and biological activities assay were employed to provide a sufficient basis for their practical applications.

**Results:**

In plant metabolomics, the PCA and OPLS-DA score plot indicated the obvious differences in chemical profiling between BC and ITM and 6 compounds were successfully identified to contribute to the differences. In DRS study, the fingerprints of 10 and 8 compounds in BC and ITM were developed based on DRS analyzer, respectively, involving relative retention time (RRT) method and linear calibration using two reference substances (LCTRS) technique. The DRS analyzer also accurately identified 10 and 8 compounds from BC and ITM, respectively, by using only two reference standards. In biological activities assay, BC had a better anticancer effect than ITM due to the high abundance of irigenin, while ITM showed stronger hepatoprotective activity than BC because of the high abundance of tectoridin.

**Conclusions:**

Therefore, due to the significant differences of *B. chinensis* and *I. dichotoma* in chemical composition and biological activities, the current studies strongly proved that these two medicinal plants could not be mixed in industrial production and clinical medication.

**Supplementary Information:**

The online version contains supplementary material available at 10.1186/s13020-021-00494-3.

## Background

*Belamcanda chinensis* (L.) DC. (BC), a perennial herbaceous plant whose rhizome is named as *She-gan* in a traditional Chinese medicine (TCM) belongs to the family of *Iridaceae* and is widely cultivated in China, Korea, Japan, India and eastern Russia as an economic medicinal plant. *She-gan* has been used in many Chinese patent medicine and Chinese medicinal formulae such as Xiaoer Qingre granules, Xiaoer Qingfei oral liquid, and Shengan Liyan oral liquid, etc., for the treatment coughing and pharyngitis.

However, *Chuan-she-gan*, the rhizome of *Iris tectorum* Maxim (ITM), another medicinal plant comes from the family of *Iridacae* mainly distributing in Sichuan of China, is also used for the treatment of asthma, cough, tonsillitis and pharyngitis. Actually, both of these two medicinal plants are rich in isoflavonoids, stilbenes, xanthones, and simple phenols [1–3]. Among them, isoflavones, such as tectoridin, iridin, tectorigenin, irigenin, irisflorentin and so on [3–5], are the major bioactive constituents of two medicines that have shown a wide range of biological activity, such as anticancer, hepatoprotective, antiatherosclerosis, antiosteoporosis and antihyperlipidemic, etc. [1, 6–8].

Consequently, in the medicinal market, as well as in the pharmaceutical industry, because of the high similarities in appearance such as color and shape between BC and ITM, they were often confused or even mixed with each other when used for the treatments of coughing and pharyngitis. Therefore, it is very necessary to distinguish these two plants from their chemical constituents and biological activities by multiple technologies and approaches, so as to provide a sufficient basis and guidance for their industrial production and clinical medication.

Metabolomics, genomics, and proteomics are important components of system biology. As an important branch of metabolomics, plant metabolomics describes the alterations in the content and composition of different plant phytochemicals [9]. Currently, the most promising technique in terms of metabolome coverage is ultra-high-performance liquid chromatography-high resolution tandem mass spectrometry (UHPLC-HRMS/MS), which is a powerful analytical tool for the analysis of the known compounds and elucidation of unknown compounds in herbal medicines. Here, UHPLC-Q Exactive-HRMS/MS based untargeted metabolomics techniques were used for qualitative studies to distinguish BC and ITM.

Quality control analysis of TCM is important for safe and effective use. The reference standard is the most important role for qualitative and quantitative analysis of TCM. There is an increasing demand for reference standards with the development of TCM quality control. At the same time, some TCM compounds are difficult to be extracted, isolated, and purified, led to a significant increase in the cost of TCM analysis. Linear calibration using two reference substances (LCTRS) approaches, a substitute reference standard method, can deal with the above problems effectively [10–12]. LCTRS is a method for the qualitative determination of several compounds to be measured by two reference standards by using several constant eigenvalues and algorithms. The principle of LCTRS is that there is a linear relationship between the retention time (t_R_) of the compounds on two different HPLC systems (including chromatographs and columns). The method has been successfully developed for the quality analysis of *Salvia miltiorrhiza*, *Paris polyphylla*, *Rheum officinale* [10–12]. Relative retention time (RRT) technique, a qualitative substitute reference standard method, can qualitative determination of several compounds to be measured by one reference standard. Finally, we introduced the concept of the digital reference standard (DRS), which supports the chromatographic algorithm methods of RRT and LCTRS. In the present study, quality control methods of fingerprint involving 10 compounds of BC and 8 compounds of ITM respectively were developed based on DRS method.

Recently, several studies on the quality control of BC were reported. Li et al. [13] evaluated the quality of BC by the establishment of chromatographic fingerprinting profile employing HPLC–DAD-MS method and simultaneous determination of seven phenol compounds. Chen et al. [14] reported the spatial chemical profiles of BC at different growth ages from various origins through qualitative and quantitative analyses by using UHPLC-Q/TOF–MS and UHPLC-QqQ-MS. Wen et al. [15] used a chemical profiling method to evaluate the quality of BC and compare the chemical compositions by HPLC analysis combining with multivariate data analysis. However, these methods only focused on one or several marker compounds and failed to distinguish these two plants from their chemical constituents and biological activities. Herein, the systematic studies on the chemical constituents, qualities and biological activities of BC and ITM were carried out by plant metabolomics, digital reference standard analyzer and biological activities assay.

## Methods

### Collection of plant materials

The rhizomes of two original medicinal plants of *Belamcanda chinensis* (L.) DC and *Iris tectorum* Maxim were collected from different habitats in China, and dried at room temperature. All of them were identified by Professor Yi Zhang. Their sample number, species, habitats and collection time were listed in Table 1.

**Table 1:** The sorts of *Belamcanda chinensis* (L) DC and *Iris tectorum* Maxim

No.	Species	Location	Collection time
BC01	*B. chineses*	Anguo, Hebei	April 2020
BC02	*B. chineses*	Baoding, Hebei	April 2020
BC03	*B. chineses*	Yuncheng, Henan	April 2020
BC04	*B. chineses*	Qinhuangdao, Hebei	April 2020
BC05	*B. chineses*	Nangong, Hebei	May 2020
BC06	*B. chineses*	Yuncheng, Shanxi	May 2020
ITM01	*I. tectorum*	Chengdu, Sichuan	April 2020
ITM02	*I. tectorum*	Chengdu, Sichuan	April 2020
ITM03	*I. tectorum*	Chengdu, Sichuan	May 2020
ITM04	*I. tectorum*	Chengdu, Sichuan	May 2020
ITM05	*I. tectorum*	Baoding, Hebei	May 2020
ITM06	*I. tectorum*	Guiyang, Guizhou	April 2020
ITM07	*I. tectorum*	Bozhou, Anhui	April 2020

### Chemicals and reagents

Chromatography-grade acetonitrile and methanol were purchased from Fisher Chemical (CA, USA). MS-grade ammonium acetate, formic acid, acetonitrile and methanol were obtained from Fisher Chemical (CA, USA). Chromatography grade phosphoric acid and ethanol were purchased from Sinopharm Chemical Reagent (Shanghai, China). Methyl-β-cyclodextrin (Me-β-CD) was obtained from Aladdin Co., Ltd (Shanghai, China). The water used was produced by Milli-Q system (Millipore, Bedford, MA, USA). Tectoridin, bicyclol and irisflorentine were purchased from National Institutes for Food and Drug Control (NIFDC, Beijing, China). Iristectorin A and iristectorigenin-A-7-glucoside were obtained from Nature Standard Technical Service Co., Ltd (Shanghai, China). Irigenin 7-glucoside was purchased from Chengdu Push Bio-technology Co., Ltd (Chengdu, China). Tectorigenin was obtained from Chengdu Puruifa Technology Co., Ltd (Chengdu, China). Iristectorigenin B, 3′,6-dimethoxy-4′,5,7-trihydroxyisoflavone, 5,7-dihydroxy-3-(3-hydroxy-4,5-dimethoxyphenyl)-6-methoxy-4-benzopyrone and 5,3-dihydroxy-4,5-dimethoxy-6,7-methylenedioxyisoflavone were purchased from Shanghai Tongtian Biotechnology Co., Ltd (Shanghai, China). Oxaliplatin was obtained from Oxaliplatin, Hospira Inc., (IL, Australia). d-galactosamine, curcumin, cell counting kit-8, RPMI1640 medium and DMEM high glucose culture medium were purchased from Meilun Biotechnology Co., Ltd (Dalian, China).

### Standard solutions and sample preparation for LC‑HR/MS metabolomics and DRS study

The dry powder (0.1 g) of BC and ITM were extracted with 25 mL of 70% ethanol in an ultrasonic bath for 60 min. The supernatant was filtered through a 0.22 μm membrane filter before analysis. All reference chemicals were dissolved in 70% ethanol at 1.0 mg mL^−1^ as stock solutions. These stock solutions were stable for at least 1 week at room temperature. A defined amount of the above stock solutions were mixed and diluted to an appropriate concentration as the standard stock solution: these standards were stable at least for 2 weeks under 4 °C.

### Plant metabolomics with liquid chromatography/high‑resolution mass spectrometry (LC‑HR/MS) analysis

The analyses were performed on an Ultimate 3000 UHPLC system coupled to Q Exactive MS (Thermo Fisher Scientific, CA, USA). UHPLC analyses were performed on a Waters Acquity UPLC BEH C_18_ column (2.1 × 100 mm, 1.7 μm; Waters Technologies, MA, USA) and Waters Van Guard BEH C_18_ column (2.1 × 5 mm, 1.7 μm; Waters Technologies, MA, USA). The mobile phase was consisted of (A) 0.1% formic acid–water and (B) acetonitrile, and the gradient program was optimized as follows: 0–1 min, 5% B; 1–9 min, 5–25% B; 9–19 min, 25–75% B; 19–25 min, 75–100% B; 25–26 min 100–5% B, 26–30 min 5% B. The column temperature was set at 45 °C. The injection volume was 1 μL. The flow rate was set at 0.4 mL min^−1^. The MS analysis of lipids was carried out on Q Exactive under the conditions: full MS/ddMS^2^ mode; evaporation temperature, 350 °C; capillary temperature, 320 °C; spray voltage, 3.0 kV for negative ion mode and 3.5 kV for positive ion mode; aux gas flow rate (arb), 10; sheath gas rate (arb), 35; mass range (*m/z*), 100–1500.

### DRS study with instruments and chromatographic conditions

Chromatographic analysis was performed on Agilent 1260 high-performance liquid chromatography with a DAD detector (Agilent Technologies, CA, USA) and Waters e2695 high-performance liquid chromatography with a 2998 PDA detector (Waters Technologies, MA, USA). Twenty-four columns (Table 2) from mainstream manufacturers were randomly selected. DRS method research is recommended to use at least ten columns from three manufacturers.

**Table 2:** Information of columns

No.	Brand	Type	Specification
Column 1	Osaka Soda	Capcell pak C_18_	250 × 4.6 mm, 5 μm
Column 2	Dikma	Diamonsil C_18_	250 × 4.6 mm, 5 μm
Column 3	Phenomenex	Luna C_18_	250 × 4.6 mm, 5 μm
Column 4	TechMate	TechMate C_18_	250 × 4.6 mm, 5 μm
Column 5	FLM	Titank C_18_	250 × 4.6 mm, 5 μm
Column 6	Waters	Symmetry C_18_	250 × 4.6 mm, 5 μm
Column 7	Waters	SunFire C_18_	250 × 4.6 mm, 5 μm
Column 8	AkzoNobel	Kromasil 100-5-C_18_	250 × 4.6 mm, 5 μm
Column 9	Agilent	ZORBAX Eclipse Plus C_18_	250 × 4.6 mm, 5 μm
Column 10	SHIMADZU	Shim-pack GIST C_18_	250 × 4.6 mm, 5 μm
Column 11	Exmere Ltd	Exsil Mono 100 C_18_	250 × 4.6 mm, 5 μm
Column 12	SHIMADZU GL	Inertsil ODS-3 C_18_	250 × 4.6 mm, 5 μm
Column 13	Dikma	Inspire C_18_	250 × 4.6 mm, 5 μm
Column 14	Agilent	ZORBAX Eclipse XDB C_18_	250 × 4.6 mm, 5 μm
Column 15	Agilent	ZORBAX SB C_18_	250 × 4.6 mm, 5 μm
Column 16	Agilent	5 HC C_18_	250 × 4.6 mm, 5 μm
Column 17	Agilent	5 TC C_18_	250 × 4.6 mm, 5 μm
Column 18	YMC	Pack ODS-A	250 × 4.6 mm, 5 μm
Column 19	ZHONGPU	RP-C_18_	250 × 4.6 mm, 5 μm
Column 20	SVEA	C_18_ Opal	250 × 4.6 mm, 5 μm

The main constituents of *B. chinensis* and *I. tectorum* are iristectorigenin A, iristectorigenin B, and irigenin. Because of the high similarity in their chemical constituents (Appendix Fig. 8), it is difficult to separate these three compounds by conventional HPLC method. Previous study found that methyl-*β*-cyclodextrin (Me-*β*-CD) could successfully resolve these problems and the resolution of the other components could also meet the content determination requirements [16]. Thus, Me-β-CD is used to the mobile phase additive. Mobile phase A was acetonitrile, mobile phase B was 0.1% phosphoric acid and 0.55% Me-β-CD-water and mobile phase C was 0.1% phosphoric acid–water. The elution procedure was shown in Table 3. The detection wavelength was 266 nm, and the UV–Vis absorption spectra (210–600 nm) were collected. The column temperature was 35 °C, the flow rate was 1 mL min^−1^, and the injection volume was 10 μL.

**Table 3:** The gradient elution conditions of the mobile phase

Time (min)	Phase A (%)	Phase B (%)	Phase C (%)
0–15	5–17	0–83	95–0
15–24	17–20	83–80	0
24–48	20–24	80–76	0
48–52	24–28	76–72	0
52–60	28–31	72–69	0
60–65	31–35	69–65	0
65–80	35–70	65–30	0
80–81	70–5	30–0	0–95
81–90	5	0	95

### Data processing

The raw LC-HRMS data files (.raw) were uploaded to the XCMS web version platform (https://xcmsonline.scripps.edu/) for retention time alignment, peak picking, and annotation [17]. The chromatographic peak data were normalized uniformly, and the multidimensional data were further analyzed by the SIMCA-P software 14.1 (Umetrics, Umea, Sweden) for multivariate data analysis by principal components analysis (PCA), orthogonal partial least squares-discriminant analysis (OPLS-DA).

### Evaluation of in vitro bioactivities

All compounds (tectoridin, iridin, tectorigenin, iristectorigenin B, iristectorigenin A, irigenin, irisflorentine, dichotomitin), BC 70% ethanol extract and ITM 70% ethanol extract were evaluated for their cytotoxicity against HepG2 and A549 cell lines using cell counting kit-8 (CCK-8) methods. Simultaneously, all standard compounds and ethanol extracts were evaluated for their hepatoprotective activities in BRL-3A and L02 cell lines as well as neuroprotective activities in BV2 cells in vitro.

### Cell culture

HepG2, A549, BRL-3A, L02 and BV2 cell lines were obtained from Shenyang Pharmaceutical University. Cells were cultured in appropriate medium (DMEM or RPMI1640) supplemented with 10% FBS, 1% penicillin/streptomycin and 5% CO_2_ at 37 °C.

### Toxicity measurements

The cytotoxicity against HepG2 and A549 cells of 8 compounds, BC and ITM ethanol extracts were determined by CCK-8 assay. Briefly, 1 × 104 cells were placed into each well of a 96-well plate and preincubated for 24 h in cell culture incubator (37 °C, 5% CO_2_). Secondly, cells were treated with different concentrations of 8 compounds (100–0.7813 μM), BC and ITM ethanol extracts (6400–25 μg mL^−1^) for 24 h, respectively. Then, cell viability was measured by cell counting kit-8 (CCK-8) assay (Dalian Meilun Biotechnology, Dalian, China) according to the manufacturer’s instructions. The absorbance values at 450 nm (OD450) were observed by Tecan Spark 10 K microplate reader (Tecan, Mannedorf, Switzerland). The viability of the control group is defined as 100%. Oxaliplatin was tested as a positive control.

### Hepatoprotective and neuroprotective assay

Eight compounds and 2 ethanol extracts were evaluated for their hepatoprotective activities against d-galactosamine induced L02 cell injury in vitro. The neuroprotective effects were determined by BV2 cells. The cells were maintained in an appropriate medium (DMEM or RPMI1640, Dalian Meilun Biotechnology, Dalian, China) in a humidified atmosphere of 5% CO_2_ at 37 °C. The cells were seeded into 96-well plates at a density of 1 × 10^5^ cells mL^−1^. After attachment, the cells were pretreated with the test compounds and extracts in different concentrations for 2 h. d-galactosamine (25 mM) or LPS solution (10 μg mL^−1^) was subsequently added for incubating another 24 h. Then, cell viability was measured by cell counting kit-8 (CCK-8) assay (Dalian Meilun Biotechnology, Dalian, China) according to the manufacturer’s instructions. The absorbance values at 450 nm (OD450) were observed by Tecan Spark 10 K microplate reader (Tecan, Mannedorf, Switzerland). The viability of the control group is defined as 100%. Bicyclol and curcumin was tested as a positive control.

$${\text{Relative survival rate \%}}=[{\rm OD} {(\rm sample)}-{\mathrm{OD}}({\mathrm{control}})/{\mathrm{ OD}}({\mathrm{normal}}))-{\mathrm{OD}}({\mathrm{control}})]\times 100{\%}.$$

### Statistical analysis

All the data are presented as mean ± SD. The level of significance between the two groups was analyzed by Students’ test, more than two groups were assessed by one-way or two-way analysis of variance (ANOVA) followed by Tukey’s multiple comparison’s test. Statistical analysis was performed using SPSS 19.0 (IBM, Chicago, IL, USA). All the results were considered statistically significant at *P* < 0.05.

## Results

### Phytochemical analysis by LC–MS/MS in BC and ITM

**Fig. 1:**
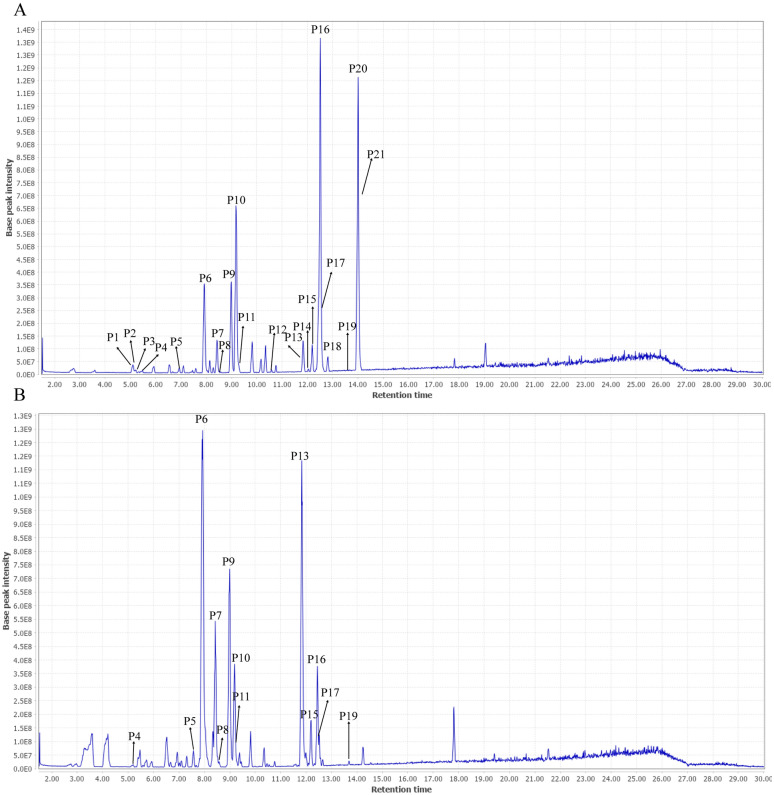
UHPLC-Q-Exactive MS base peak intensity (BPI) chromatogram of BC (**A**) and ITM (**B**) in positive ion mode

**Fig. 2:**
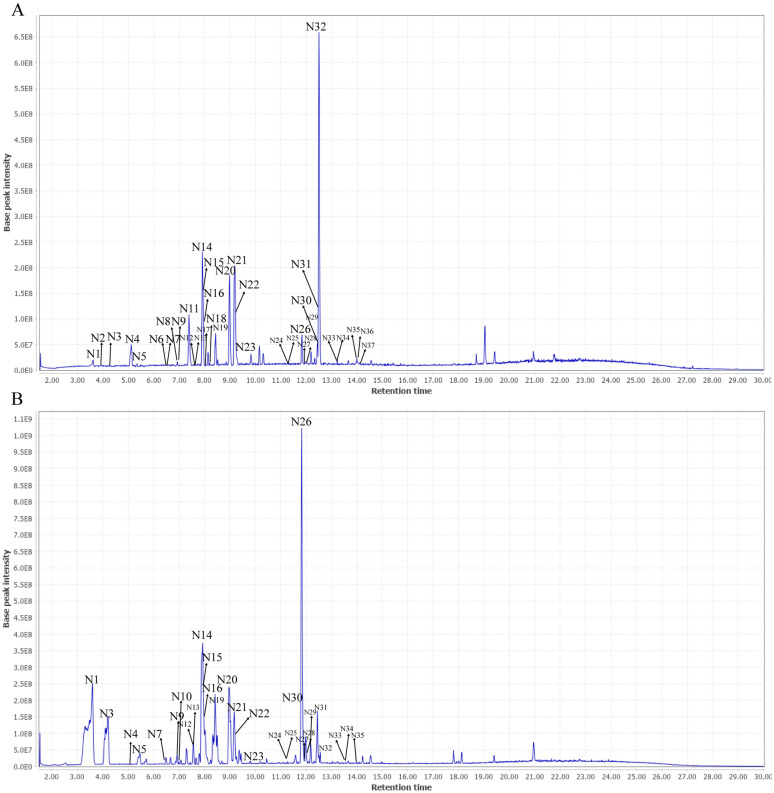
UHPLC-Q-Exactive MS BPI chromatogram of BC (**A**) and ITM (**B**) in negative ion mode

UHPLC-Q Exactive-MS/MS was conducted in both positive and negative ion mode to explore the phytochemical identification of BC and ITM extracts. The retention time, accurate molecular weight, quasi-molecular, formula and MS/MS fragments observed in the positive and negative mode were summarized in Tables 4 and 5. In present study, 40 compounds, including 25 isoflavone glycosides, 5 xanthones, 1 flavone and 9 other compounds were unambiguously or tentatively identified (Figs. 1, 2). By comparing with authentic standards and MS spectra, 10 compounds, including tectoridin, iristectorin A, iristectorin B, iridin, tectorigenin, iristectorigenin B, iristectorigenin A, irigenin, irisflorentine and dichotomitin were identified definitely, respectively (Additional file 1: Tables S1–S3).

**Table 4:** Chemical characterization of *B. chinensis* (BC) and *I. tectorum* (ITM) by UHPLC-Q-exactive-MS/MS in positive ion

Peaks	Retention time (min)	*m/z* Quasi-molecular [M + H]^+^	*m/z* Calculated [M + H]^+^	Error (ppm)	Formula	MS/MS fragments	Identification	Reference	Source
P1	5.13	423.0919	423.0922	− 7.09	C_19_H_18_O_11_	405, 333, 305, 303, 275	Mangiferin	[14, 18–20]	BC, ITM
P2	5.35	423.0919	423.0922	− 7.09	C_19_H_18_O_11_	405, 333, 305, 303, 275	Isomangiferin	[14, 18, 20]	BC, ITM
P3	5.35	437.1087	437.1078	20.59	C_20_H_20_O_11_	315, 303, 301, 279, 227	7-*O*-Methylmangiferin	[14, 18]	BC
P4	5.49	625.1762	625.1763	− 1.60	C_28_H_32_O_16_	463, 340, 303, 279, 227	Tectorigenin-7-*O*-glucosyl-4'-*O*-glucoside	[14, 18, 19, 21]	BC, ITM
P5	6.95	625.1760	625.1763	− 4.80	C_28_H_32_O_16_	540, 510, 463, 437, 315, 301	Tectorigenin-7-*O*-*β*-glucosyl (1–6) glucoside	[14, 18, 20]	BC, ITM
P6	7.93	463.1232	463.1235	− 6.48	C_22_H_22_O_11_	301, 286	Tectoridin^a^	[14, 18–20]	BC, ITM
P7	8.45	493.1345	493.1341	8.11	C_23_H_24_O_12_	463, 331, 316, 307, 229	Iristectorin A^a^	[14, 18, 20]	BC, ITM
P8	8.52	479.119	479.1184	12.52	C_22_H_22_O_12_	331, 317, 287	3′-Hydroxytectoridin	[14, 18, 20]	BC, ITM
P9	8.99	493.1347	493.1341	12.17	C_23_H_24_O_12_	331, 314, 279, 261, 199, 154	Iristectorin B^a^	[14, 18–20]	BC, ITM
P10	9.19	523.1381	523.1373	15.29	C_24_H_26_O_13_	361, 340	Iridin^a^	[14, 18, 20]	BC, ITM
P11	9.19	523.1441	523.1446	− 9.56	C_24_H_26_O_13_	361, 340, 279, 227, 199, 154, 142	Iridinisomer	[14, 18, 20]	BC, ITM
P12	10.6	535.1435	535.1446	− 20.56	C_25_H_26_O_13_	421, 377, 336, 315	3′,5′-Dimethoxyirisolone-4′-*O*-*β*-d-glucoside	[14, 18, 20]	BC
P13	11.84	301.0706	301.0707	− 3.32	C_16_H_12_O_6_	286, 231, 154, 142	Tectorigenin^a^	[14, 18, 20]	BC, ITM
P14	12.05	673.1777	673.1763	20.80	C_32_H_32_O_16_	643, 615, 515, 361, 301	6′′-*O*-vanilloyliridin	[14, 18]	BC
P15	12.18	331.0798	331.0812	− 42.29	C_17_H_14_O_7_	316, 303, 279, 254, 234	Iristectorigenin B^a^	[14, 18–20]	BC, ITM
P16	12.43	331.0796	331.0812	− 48.33	C_17_H_14_O_7_	316, 303, 279, 254, 234	Iristectorigenin A^a^	[14, 18, 20]	BC, ITM
P17	12.51	361.0912	361.0918	− 16.62	C_18_H_16_O_8_	346, 331, 183	Irigenin^a^	[14, 18, 20]	BC, ITM
P18	12.82	373.0919	373.0918	2.68	C_19_H_16_O_8_	361, 331, 301, 279, 226	Noririsflorentin	[14, 18]	BC
P19	13.69	299.0549	299.055	− 3.34	C_16_H_10_O_6_	228, 199, 169, 154	Irilone	[14, 18–19]	BC, ITM
P20	14.00	387.1071	387.1074	− 7.75	C_20_H_18_O_8_	359, 329, 262	Irisflorentin^a^	[14, 18–20]	BC
P21	14.06	359.0765	359.0761	11.14	C_18_H_14_O_8_	329, 299, 271, 248, 223	Dichotomitin^a^	[14, 18–20]	BC

^a^Authentic standards

**Table 5:** Chemical characterization of *B. chinensis* (BC) and *I. tectorum* (ITM) by UHPLC-Q-exactive-MS/MS in negative ion

Peaks	Retention time (min)	*m/z* Quasi-molecular [M-H]^−^	*m/z* Calculated [M-H]^−^	Error (ppm)	Formula	MS/MS fragments	Identification	Reference	Source
N1	3.6	535.166	535.1668	− 14.95	C_21_H_30_O_13_	525, 489, 323, 235, 215	Tectoruside	[3, 19]	BC, ITM
N2	3.96	583.1314	583.1305	15.43	C_25_H_28_O_16_	535, 489, 403, 352, 307, 273	Neomangiferin	[3, 18, 19]	BC
N3	4.23	373.1141	373.114	2.68	C_15_H_20_O_8_	363, 273, 235, 215	Androsin	[3, 19]	BC, ITM
N4	5.12	421.078	421.0776	9.50	C_19_H_18_O_11_	364, 327, 307, 273, 235, 215	Mangiferin	[3, 18, 19]	BC, ITM
N5	5.35	421.0779	421.0776	7.12	C_19_H_18_O_11_	395, 333, 307, 273, 255, 235, 215	Isomangiferin	[3, 18]	BC, ITM
N6	6.40	435.0923	435.0933	− 22.98	C_20_H_20_O_11_	377, 339, 307, 275, 235, 215	7-*O*-methylmangiferin	[3, 18]	BC
N7	6.50	447.0926	447.0933	− 15.66	C_21_H_20_O_11_	327, 313, 285, 235, 215	Luteolin-6-C-*β*-d-glucoside	[3, 22]	BC, ITM
N8	6.93	435.0925	435.0933	− 18.39	C_20_H_20_O_11_	391, 352, 313, 275, 235, 215	7-*O*-methylisomangiferin	[3, 18]	BC
N9	6.95	623.1622	623.1618	6.42	C_28_H_32_O_16_	567, 537, 435, 313, 299, 284, 235,215	Tectorigenin-7-*O*-glucosyl-4′-*O*-glucoside	[3, 23]	BC, ITM
N10	7.11	463.1246	463.1246	0.00	C_22_H_24_O_11_	327, 273, 235, 215	Dihydrokaempferol-7-*O*-glucoside	[3, 23]	ITM
N11	7.37	653.1712	653.1723	− 16.84	C_29_H_34_O_17_	595, 509, 403, 329, 243	Iristectorigenin-A-7-*O*-*β*-glucosyl (1 → 6)-glucoside	[3, 18, 19]	BC
N12	7.57	431.0973	431.0984	− 25.52	C_21_H_20_O_10_	431, 269, 235, 215	Saponaretin	[3, 22]	BC, ITM
N13	7.57	477.1029	477.1038	− 18.86	C_21_H_20_O_10_	431, 269, 235	Genistein-7-*O*-glucoside	[3, 24]	BC, ITM
N14	7.92	461.1087	461.1089	− 4.34	C_22_H_22_O_11_	299	Tectoridin^a^	[3, 18]	BC, ITM
N15	7.92	461.1087	461.1089	− 4.34	C_22_H_22_O_11_	413, 352, 329, 299, 284, 255	Tectorigenin-4′-*O*-*β*-d-glucoside	[3, 18]	BC, ITM
N16	7.92	507.1147	507.1144	5.92	C22H22O11	461, 299	Isotectorigenin-7-*O*-*β*-d-glucoside	[3, 18]	BC, ITM
N17	8.01	593.1499	593.1512	− 21.92	C_27_H_30_O_15_	507, 461, 299	Genistein-7-Ogentiobioside	[3, 25]	BC
N18	8.21	447.0925	447.0933	− 17.89	C_21_H_20_O_11_	352, 317, 273, 235, 215	Orobol-7-*O*-d-glucoside	[3, 24]	BC
N19	8.43	491.1202	491.1195	14.25	C_23_H_24_O_12_	329, 314, 227, 215	Iristectorin A^a^	[3, 18, 19]	BC, ITM
N20	8.99	491.1201	491.1195	12.22	C_23_H_24_O_12_	329, 314, 227, 215	Iristectorin B^a^	[3, 18, 19]	BC, ITM
N21	9.19	521.1293	521.1301	− 15.35	C_24_H_26_O_13_	427, 359, 344	Iridin^a^	[3, 18]	BC, ITM
N22	9.19	567.1348	567.1355	− 12.34	C_24_H_26_O_13_	521, 507, 359, 344	Isoiridin	[3, 18]	BC, ITM
N23	9.83	257.0815	257.0819	− 15.56	C_15_H_14_O_4_	246, 230, 215	Gnetucleistol D	[3]	BC, ITM
N24	11.57	653.1708	653.1723	− 22.96	C_29_H_34_O_17_	326, 299, 269	Iristectorin-B-4′-*O*-glucoside	[3, 18]	ITM
N25	11.68	519.113	519.1144	− 26.97	C_24_H_24_O_13_	326, 268, 230	Dichotomitin-3′-*O*-glucoside	[3, 18, 19]	BC, ITM
N26	11.84	299.0557	299.0561	− 13.38	C_16_H_12_O_6_	284, 268, 242, 230, 215, 195	Tectorigenin^a^	[3, 18, 19]	BC, ITM
N27	12.02	641.151	641.1512	− 3.12	C_31_H_30_O_15_	459, 299	6′′-*O*-phydroxybenzoyliridin	[3, 18]	BC, ITM
N28	12.04	671.1599	671.1618	− 28.31	C_32_H_32_O_16_	641, 613, 359, 326, 299	6′′-*O*-vanilloyliridin	[3, 18]	BC, ITM
N29	12.18	329.0667	329.0667	0.00	C_17_H_14_O_7_	315, 286, 268, 242	Iristectorigenin B^a^	[3, 18, 19]	BC, ITM
N30	12.43	329.0667	329.0667	0.00	C_17_H_14_O_7_	315, 286, 268, 242, 198	Iristectorigenin A^a^	[3, 18]	BC, ITM
N31	12.46	301.0715	301.0718	− 9.96	C_16_H_14_O_6_	280, 273, 242, 215	Dihydrokaempferide	[3, 23]	BC, ITM
N32	12.51	359.0763	359.0772	− 25.06	C_18_H_16_O_8_	344, 329, 181	Irigenin^a^	[3, 18]	BC, ITM
N33	13.66	297.0406	297.0405	3.37	C_16_H_10_O_6_	280, 258, 230, 215	Irilone	[3, 18, 19]	BC, ITM
N34	13.68	343.0822	343.0823	− 2.91	C_18_H_16_O_7_	318, 297, 280	Dalspinosin	[3]	BC, ITM
N35	13.94	327.0511	327.051	3.06	C_17_H_12_O_7_	294, 258, 230	Iriflogenin	[3]	BC, ITM
N36	13.94	373.0919	373.0929	− 26.80	C_19_H_18_O_8_	336, 294, 280, 258, 230, 215	Junipegenin C	[3, 18]	BC
N37	14.04	357.0607	357.0616	− 25.21	C_18_H_14_O_8_	294, 230	Dichotomitin^a^	[3, 18, 19]	BC

^a^Authentic standards

For the MS spectra of isoflavonoid glycosides, the neutral loss of 162 Da was demonstrated as the characteristic [M + H − 162]^+^ ion and [M − H − 162]^−^ of *O*-glycosides such as P4/N9, P5, P6/N14, P7/N19, P8, P9/N20, P10/N21, N11, N15, N16, N22, N24 (Appendix Fig. 9a). Tectorigenin-7-*O*-*β*-glucosyl (1–6) glucoside and tectorigenin-7-*O*-glucosyl-4′-*O*-glucoside showed loss of 324 Da (-2glycosides), and generated m/z 301 or 299 ions corresponding to the [M + H − 2glycosyl]^+^ or [M − H − 2glycosyl]^−^ fragments which was the same as [M + H]^+^ or [M − H]^−^ ion of tectorigenin [3]. MS/MS spectra of irigenin showed successive loss of 15 Da, 30 Da and 178 Da, and generated *m/z* 346, 331 and 183 ions corresponding to the [M + H − CH_3_]^+^, [M + H − 2CH_3_]^+^ and [M + H − C_10_H_10_O_3_]^+^ fragments. The fragment ion at *m*/*z* 183 derived from irigenin Retro–Diels–Alder (RDA) fragmentation, which were observed in all the isoflavones and the most characteristic ion for these isoflavonoids [13]. This RDA fragmentation was shown in Appendix Fig. 9b. By the similar method, other isoflavone aglycones were also characterized.

In this work, five xanthones including mangiferin, isomangiferin, 7-*O*-methylmangiferin, neomangiferin, 7-*O*-methylisomangiferin were identified in the extracts of BC and ITM. Xanthones showed [M + H − 120]^+^ and [M − H − 120]^−^ fragment ions in MS/MS spectra, which were typical of xanthone C-glucosides [18]. MS/MS spectra of mangiferin showed successive loss of 18 Da and 120 Da, and generated *m/z* 405 and 303 ions corresponding to the [M + H − H_2_O]^+^ and [M + H − C_4_H_8_O_4_]^+^ fragments (Appendix Fig. 9c). P2 was tentatively assigned as an isomer of mangiferin, isomangiferin. By the similar method, neomangiferin were characterized. Compounds 7-*O*-methylmangiferin and 7-*O*-methylisomangiferin showed similar fragmentation behaviors as that of mangiferin, thus they could be deduced as derivatives of mangiferin. The [M − H]^−^ ions of 7-*O*-methylmangiferin and 7-*O*-methylisomangiferin were both observed at *m*/*z* 435, 14 Da more than that of mangiferin. It could be presumed that a hydroxyl group was replaced by a methoxy group in structures of 7-*O*-methylmangiferin and 7-*O*-methylisomangiferin [18]. This fragmentation was shown in Appendix Fig. 9.

### BC and ITM in LC‑HR/MS metabolomics

**Fig. 3:**
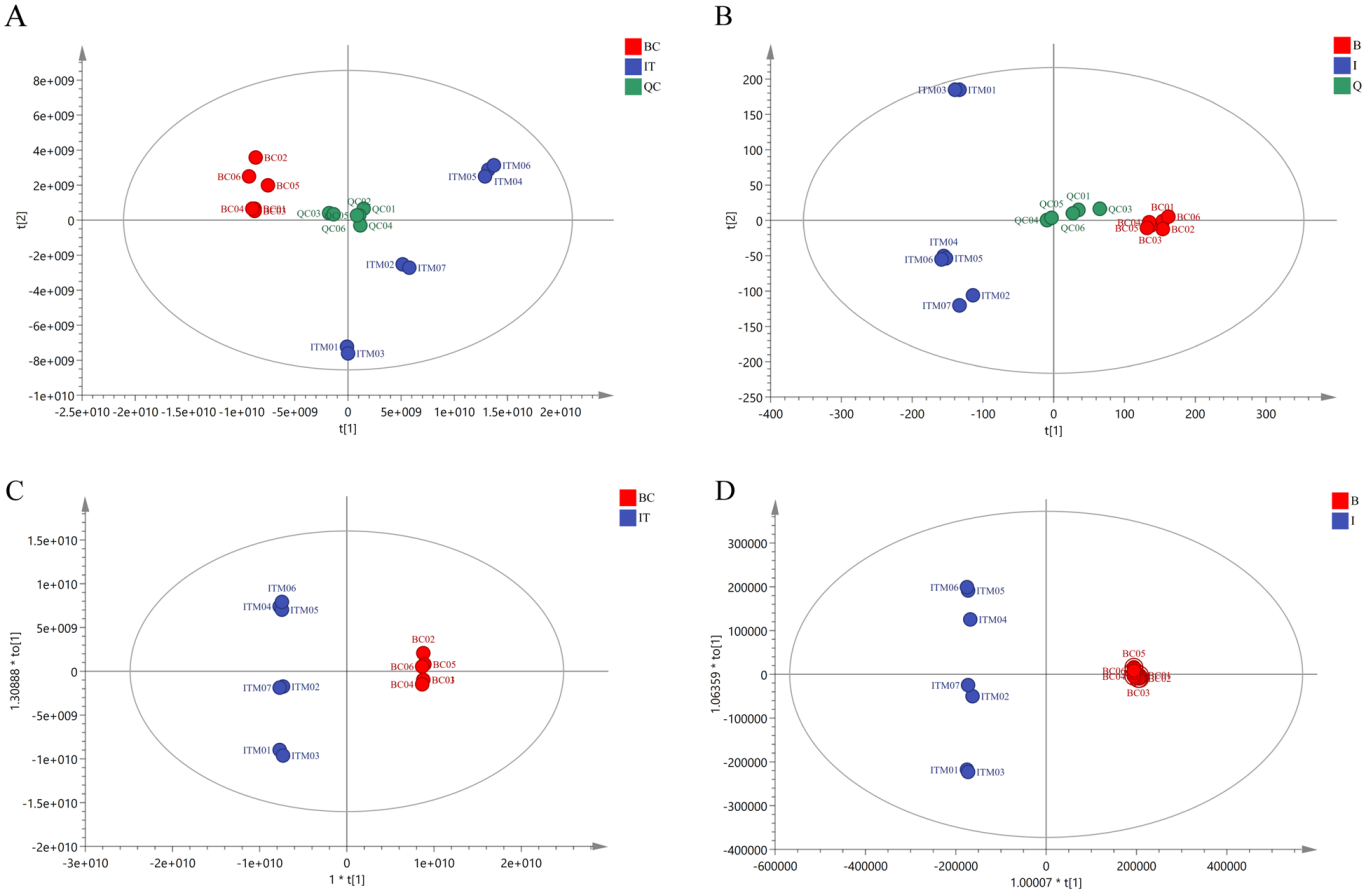
Score plots of PCA in positive ion (**A**) and negative ion (**B**). Score plots of OPLS-DA in positive ion (**C**) and negative ion (**D**) for LC-HR/MS metabolomics. Red circles: *B. chineses*; blue circles: *I. tectorum*; green circles: QC sample

**Fig. 4:**
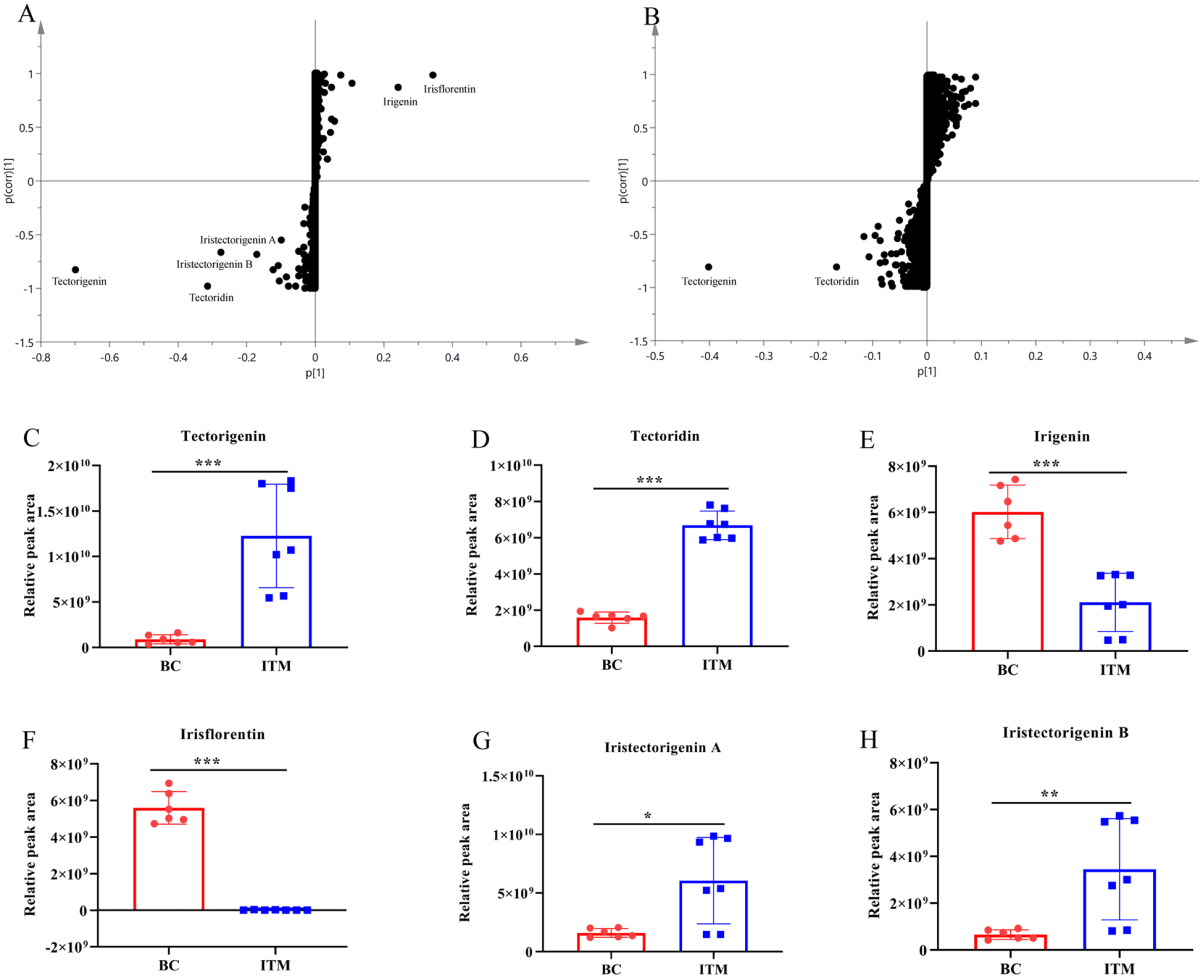
S-plots of OPLS-DA in positive ion (**A**) and negative ion (**B**) for LC-HR/MS metabolomics. (C-H) The remarkable variable of chemical constitutes

PCA was carried out using the resultant data matrix. BC (red circles) and ITM (blue circles) samples were roughly separated on the PC1 (70.2%) *vs* PC2 (11.5%) plane (Fig. 3A) in positive ion and the PC1 (27.6%) vs PC2 (10.3%) plane (Fig. 3B) in negative ion. Simultaneously, we performed OPLS-DA to search for components that could help to distinguish BC from ITM. The OPLS-DA projection models had a clearer separation of the BC and ITM samples in positive and negative ion (Fig. 3C–D). Furthermore, the s-plot indicated the following characteristic peaks: *m/z* 301.0706 (tectorigenin), *m/z* 463.1232 (tectoridin), *m/z* 361.0912 (irigenin), *m/z* 387.1071 (irisflorentin), *m/z* 331.0798 (iristectorigenin A) and *m/z* 331.0796 (iristectorigenin B) (Fig. 4A, B). These remarkable variables of chemical constitute were major phytochemical-marker between BC and ITM (Fig. 4C–H). These components were at the edge of the vertical and horizontal axes, and were explanatory variables that strongly contributed to the objective variable. The multivariate chemometric analysis demonstrated that the remarkable differences of chemical constitute between BC and ITM.

### Fingerprint analysis of BC and ITM by DRS study

#### Optimization of HPLC conditions and method validation

**Fig. 5:**
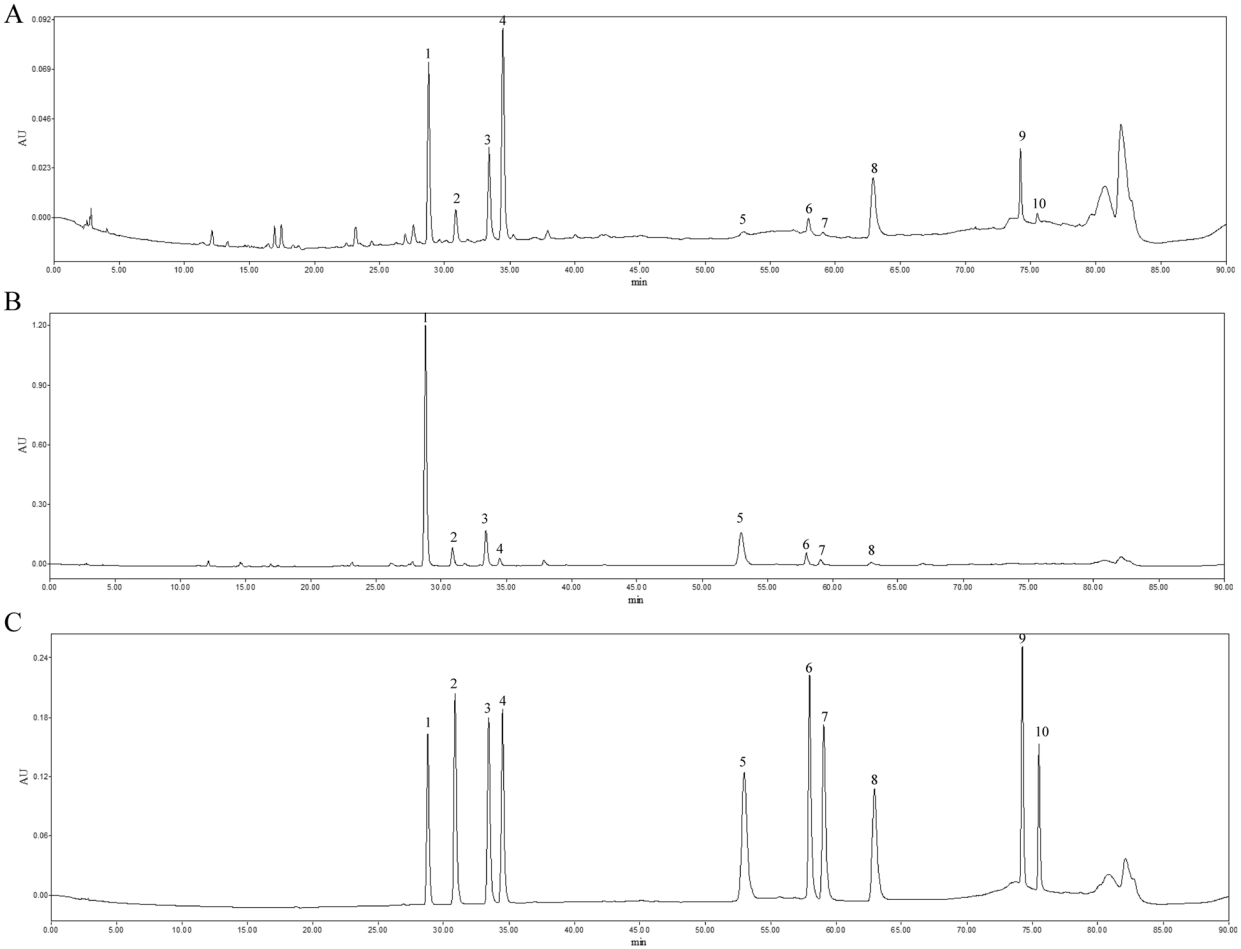
Representative HPLC chromatogram of sample on Column 12 (Exsil Mono 100 C_18_). **A** BC sample, **B** ITM sample, **C** Chromatograms of mixed standard. Tectoridin (1), Iristectorin A (2), Iristectorin B (3), Iridin (4), Tectorigenin (5), Iristectorigenin B (6), Iristectorigenin A (7), Irigenin (8), Irisflorentine (9), Dichotomitin (10)

The mobile phase, gradient elution procedures, detection wavelength and flow rates were optimized. The selected chromatographic conditions had satisfactory peak shape and resolution between peaks. Representative chromatograms and spectra were shown in Fig. 5. The peaks were identified by UV spectra and retention time (Appendix Fig. 8).

Methodological validation experiments were performed on column 14 (Inertsil ODS-3 C_18_). The precision (n = 6), stability (48 h, n = 9), and repeatability (n = 6) were tested. The results showed that RSD of the peaks t_R_ and peak areas were both less than 2%, thus meeting the requirements of fingerprint analysis.

#### Initialization for the DRS method

Details of the operating principle and applications of LCTRS were well documented in previous our literature [10–12]. In brief, the LCTRS method consisted of several steps, including data importing, peak assignment, setting the qualitative chromatographic method.

#### Optimization and evaluation of DRS method

##### Retention time prediction by LCTRS method

In our study, the St_R_ values compounds, as a reference value for retention time prediction, were determined by the arithmetic average of the retention times on nineteen columns [10–12]. In *B. chineses*, we had identified 10 compounds by standard, including tectoridin, iristectorin A, iristectorin B, iridin, tectorigenin, iristectorigenin B, iristectorigenin A, irigenin, irisflorentine and dichotomitin. Reference compounds selection is very important for the qualitative analysis in the substitute methods. According to DRS method principle, the tectoridin and dichotomitin were selected as two reference compounds for LCTRS method. The actual retention times of the 10 compounds on different columns and chromatographic instruments showed good linear relationships with their St_R_ (Table 6). Meanwhile, in *I. tectorum*, we also had identified 8 compounds by standard, including tectoridin, iristectorin A, iristectorin B, iridin, tectorigenin, iristectorigenin B, iristectorigenin A and irigenin. The tectoridin and irigenin were selected as two reference compounds for LCTRS method in *I. tectorum*, and the linear fitting results were shown in Table 6.

**Table 6:** Linear fitting results of actual retention times by LCTRS method for BC and ITM

No.	BC		ITM	
	Calibration curve	R^2^	Calibration curve	R^2^
Column 1	*Y* = 1.0425*X* − 3.9978	0.9995	*Y* = 1.0285*X* − 3.4495	0.9991
Column 2	*Y* = 0.9660*X* + 2.0604	0.9995	*Y* = 0.9480*X* + 2.6624	0.9996
Column 3	*Y* = 0.9967*X* − 0.9580	0.9994	*Y* = 0.9740*X* + 0.0858	0.9999
Column 4	*Y* = 0.9985*X* − 1.4591	0.9989	*Y* = 0.9686*X* − 0.3842	0.9994
Column 5	*Y* = 1.0189*X* − 4.5116	0.9945	*Y* = 0.9522*X* − 1.7815	0.9968
Column 6	*Y* = 0.9961*X* + 4.0136	0.9931	*Y* = 1.0885*X* + 0.7441	0.9954
Column 7	*Y* = 1.0128*X* − 0.3540	0.9997	*Y* = 1.0260*X*-0.9166	0.9997
Column 8	*Y* = 0.9829*X* + 1.1290	0.9998	*Y* = 0.9740*X* + 1.4514	0.9996
Column 9	*Y* = 0.9851*X* + 3.4759	0.9972	*Y* = 1.0386*X* + 1.5402	0.9983
Column 10	*Y* = 1.0075*X* − 1.0370	0.9999	*Y* = 0.9978*X* − 0.6472	0.9999
Column 11	*Y* = 0.9711*X*-1.1506	0.9929	*Y* = 0.8989*X* + 1.6232	0.9960
Column 12	*Y* = 1.0107*X* − 4.1726	0.9939	*Y* = 0.93393*X* − 1.2438	0.9971
Column 13	*Y* = 0.9632*X* + 3.0443	0.9997	*Y* = 0.9688*X* + 2.8581	0.9994
Column 14	*Y* = 0.9707*X* + 5.8023	0.9929	*Y* = 1.0602*X* + 2.8936	0.9959
Column 15	*Y* = 0.9838*X* + 4.0523	0.9952	*Y* = 1.0649*X* + 1.4692	0.9964
Column 16	*Y* = 1.0038*X* + 0.5351	0.9985	*Y* = 1.0407*X* − 0.8421	0.9987
Column 17	*Y* = 1.0233*X* − 2.1767	0.9999	*Y* = 1.0165*X* − 2.0256	0.9998
Column 18	*Y* = 0.9947*X* + 0.7155	0.9996	*Y* = 1.0065*X* + 0.2546	0.9994
Column 19	*Y* = 1.0340*X* − 3.6999	0.9995	*Y* = 1.0217*X* − 3.1698	0.9991

##### Retention time prediction by RRT method

The RRT method was used the single standard to identify chromatographic peaks. In *B. chineses*, we selected irigenin as the reference compound because of its appropriate retention time and availability. Meanwhile, in *I. tectorum*, we chose iristectorigenin B as the reference compound. The RRT of ten (BC) and eight (ITM) analytes relative to irigenin and iristectorigenin B were calculated by the arithmetic average of the RRTs on nineteen columns.

##### Comparison between LCTRS and RRT method

To evaluate the advantages and disadvantages of LCTRS and RRT method, we then calculated the absolute deviations (ΔtR) of the actual retention time and predicted retention time on nineteen columns (Additional file 1: Tables S4–S7). As shown in Table 7, the RRT method had a larger average deviation, a lower identification rate and available column amount than LCTRS. The above results showed that LCTRS had a precise, feasible, and superior to identify peaks than RRT.

**Table 7:** Comparison of different methods (19 columns for method establishment)

Method	Average t_R_ deviation/min	Identification rate/% (Δt_R_ ≤ 1.5 min)	Available column amount (Δt_R_ ≤ 1.5 min)
RRT (BC)	1.07	75.44	8
LCTRS (BC)	0.77	82.89	11
RRT (ITM)	0.72	84.21	10
LCTRS (ITM)	0.45	94.74	13

##### Sample tests

**Fig. 6:**
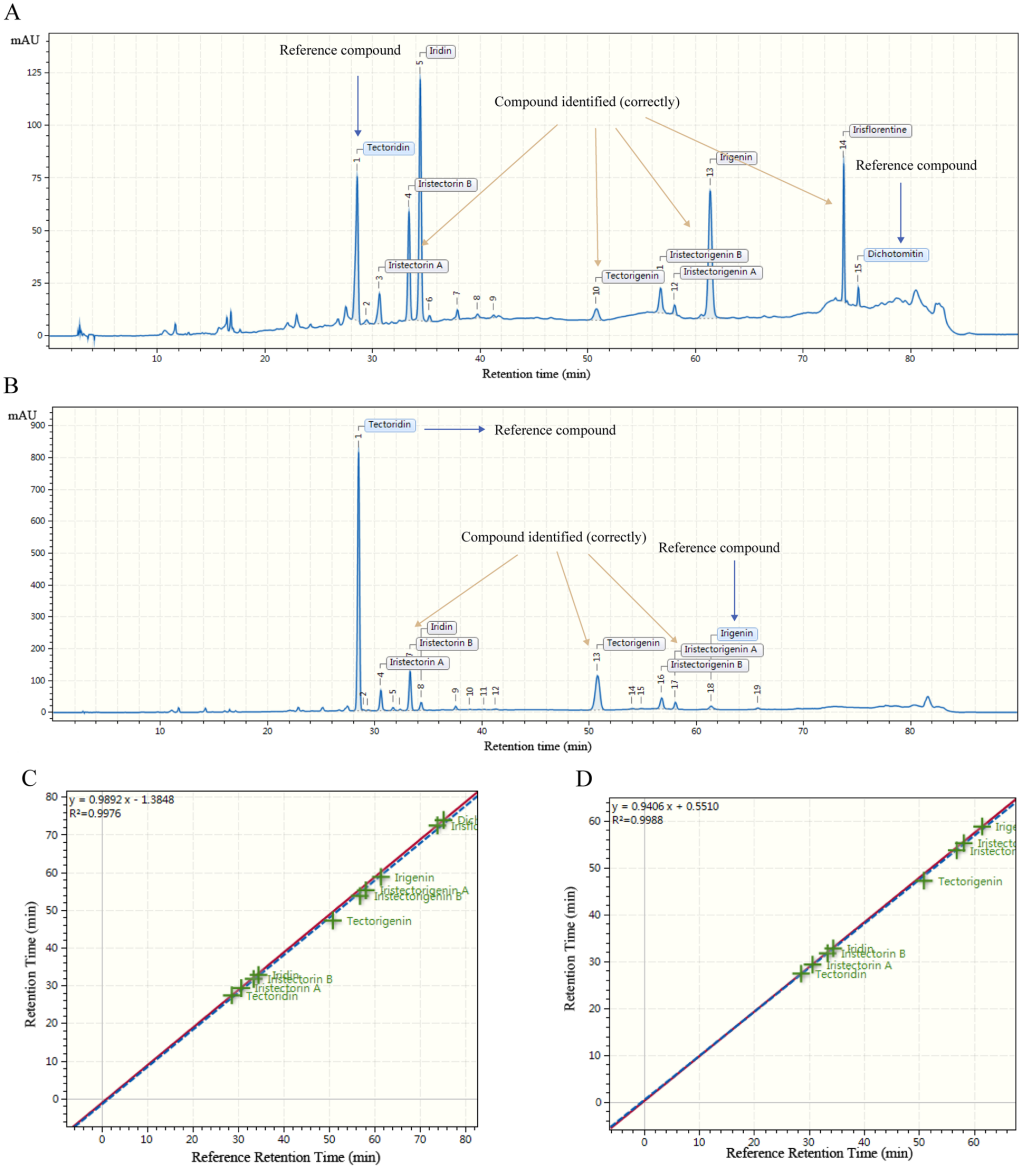
Results of sample tests on column 20 [SVEA C_18_ Opal]. **A** The result of the LCTRS method of BC. **B** The result of the LCTRS method of ITM. **C** The result of peaks and linear fitting results of BC. **D** The result of peaks and linear fitting results of ITM

To further verify the reliability of our method LCTRS, we chose SVEA C_18_ Opal column for sample testing. The detailed procedure of LCTRS method was described in our previous literature [10–12]. In brief, the LCTRS sample tests consisted of three steps, including data integrated, reference compounds assigned and the results obtained. The sample test results were exhibited in Fig. 6, which included the qualitative results of peaks and linear fitting results (Fig. 6).

### Toxicity and protective analysis

The cytotoxicity of 8 compounds (0.78–100 μM) and the extracts (25–6400 μg mL^−1^) was evaluated against HepG2 and A549 cells, respectively (Table 8). Among them, irigenin showed significantly inhibiting activity on the HepG2 cells with IC_50_ values of 18.66 µM and iristectorigenin B showed significantly inhibiting activity on the A549 cells with IC_50_ values of 35.21 µM (Table 8). Compared with BC extracts, ITM extracts showed strongly inhibitory activity against HepG2 and A549 cells with IC_50_ values of 31.41 μg mL^−1^ and 147.40 μg mL^−1^, respectively (Table 8).

**Table 8:** The toxicity of 8 compounds, BC extracts, ITM extracts and positive drug in HepG2 and A549 cells

Concentration (μM/μg mL^−1^)	HepG2 (IC_50_ ± SD)	A549(IC_50_ ± SD)
Oxaliplatin	6.02 ± 0.23	13.13 ± 0.16
Tectoridin	135.80 ± 0.63	83.98 ± 0.02
Iridin	126.20 ± 0.07	102.90 ± 0.50
Tectorigenin	60.28 ± 0.34	68.44 ± 0.33
Iristectorigenin B	38.68 ± 0.28	35.21 ± 0.22
Iristectorigenin A	44.12 ± 0.66	42.06 ± 0.02
Irigenin	18.66 ± 0.11	47.63 ± 0.44
Irisflorentine	155.70 ± 0.17	37.23 ± 0.28
Dichotomitin	161.80 ± 0.63	62.01 ± 0.11
BC extracts	31.41 ± 0.48	147.40 ± 0.57
ITM extracts	68.94 ± 0.12	366.60 ± 0.56

**Fig. 7:**
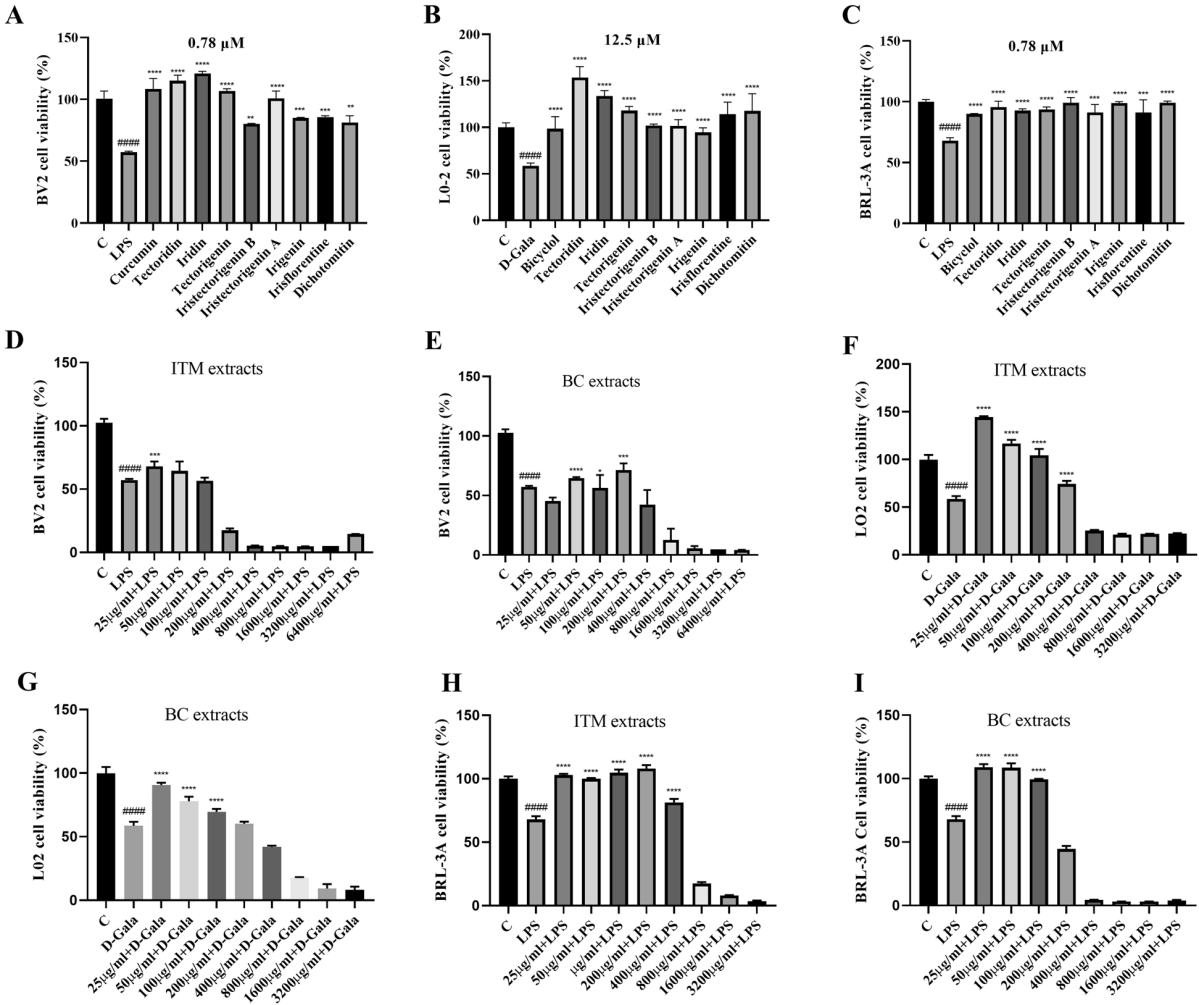
The neuroprotective and hepatoprotective of 8 compounds, BC extracts, ITM extracts and positive drug. **A**–**C** The neuroprotective and hepatoprotective of 8 compounds. **D**–**E** The neuroprotective of ITM and BC extracts in BV2 cell. **F**, **G** The hepatoprotective of ITM and BC extracts in L02 cell. **H**, **I** The hepatoprotective of ITM and BC extracts in BRL-3A cell. Cell viability was measured with a CCK-8 assay. The data are expressed as the percentage of the relative untreated control cells. All values are expressed as the mean ± SD. ^####^*P* < 0.0001 versus the control group. **P < 0.01, ***P < 0.001, ****P < 0.0001 versus the model group

To further investigate their neuroprotective and hepatoprotective, BV2 microglial cells, L02 and BRL-3A cells were treated with 8 compounds and 2 extracts (Additional file 1: Table S8). Results showed that Tectoridin and iridin at concentration of 0.75 μM exhibited higher neuroprotective activity than the positive control, curcumin (Fig. 7A, Additional file 1: Table S8). However, BC extracts and ITM extracts showed no neuroprotective effects on BV2 cells (Fig. 7D–E). Tectoridin and iridin at concentration of 12.5 μM showed more potent hepatoprotective activity than bicyclol (positive control) on L02 cells (Fig. 7B, Additional file 1: Table S8). Compared with BC extracts, ITM extracts showed more potent neuroprotective activity on L02 cells (Fig. 7F, G). Iristectorigenin B, tectoridin and irigenin showed more potent hepatoprotective activity than bicyclol on BRL-3A cells (Fig. 7C, Additional file 1: Table S8). ITM extracts showed more potent hepatoprotective activity on BRL-3A cells than BC extracts (Fig. 7H, I).

## Discussions

In our work, the plant metabolomics, digital reference standard, as well as in vitro activity assay strategy were used to evaluate the chemical constituents, qualities and biological activities of BC and ITM. In the multivariate analysis, the PCA and OPLS-DA score plot indicated the obvious differences in chemical profiling between BC and ITM. Moreover, it showed that 6 principal compounds were successfully identified to contribute to the differences in chemical profiling between BC and ITM.

In digital reference standard study, a series of quality control methods of fingerprints in BC and ITM were developed based on the DRS analyzer, involving the RRT method, LCTRS method. In BC, the tectoridin and dichotomitin were selected as two reference compounds for LCTRS method. In ITM, the tectoridin and irigenin were selected as two reference compounds for LCTRS method. The digital reference standard strategy significantly reduced the analysis cost, saved time and improved the multicomponent analysis efficiency of the analysis method.

In biological activities assay, BC has better anticancer activity than ITM due to its high abundance of irigenin. In contrast, the hepatoprotective activity of ITM was higher than that of BC because of the high abundance of tectoridin. Unfortunately, neither BC nor ITM showed good neuroprotective activity in BV2 cells.

## Conclusions

In summary, based on multidimensional strategy, it was indicated that *B. chinensis* and *I. dichotoma* were significantly different in their chemical constituents and biological activities. The results not only showed that these two medicinal plants could not be mixed in clinical medication, but also provided a novel multidimensional strategy for the identification of easily confused, easily adulterated, and even counterfeit medicinal materials.

## Supplementary Information

**Additional file 1: Table S1.** Structures of all isoflavones found in* Belamcanda chinensis* (L) DC and *Iris tectorum* Maxim. Me: CH_3_, Glu: glucose. **Table S2.** Structures of isoflavones from *Belamcanda chinensis* (L) DC and *Iris tectorum* Maximwith additional dioxolane ring. Me: CH_3_, Glc: glucose. **Table S3**. Structures of xanthonesfrom *Belamcanda chinensis* (L) DC and *Iris tectorum* Maximwith. Me: CH_3_, Glu: glucose. **Table S4**. The absolute deviations (ΔtR) of the actual retention time and predicted retention time on nineteen columns in BC LCTRS method. **Table S5**. The absolute deviations (ΔtR) of the actual retention time and predicted retention time on nineteen columns in BC RRT method. **Table S6**. The absolute deviations (ΔtR) of the actual retention time and predicted retention time on nineteen columns in ITM LCTRS method. **Table S7**. The absolute deviations (ΔtR) of the actual retention time and predicted retention time on nineteen columns in ITM RRT method. **Table S8**. Neuroprotective and hepatoprotective effects of compounds and positive drug.

## Figures and Tables

**Fig. 8:**
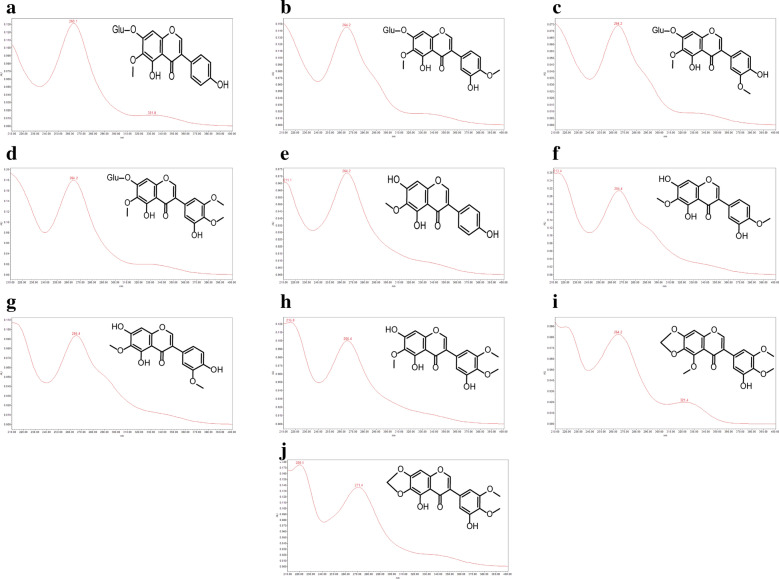
Chemical structures and UV spectra. **a** Tectoridin, **b** Iristectorin A, **c** Iristectorin B, **d** Iridin, **e** Tectorigenin, **f** Iristectorigenin B, **g** Iristectorigenin A, **h** Irigenin, **i** Irisflorentine, **j** Dichotomitin

**Fig. 9:**
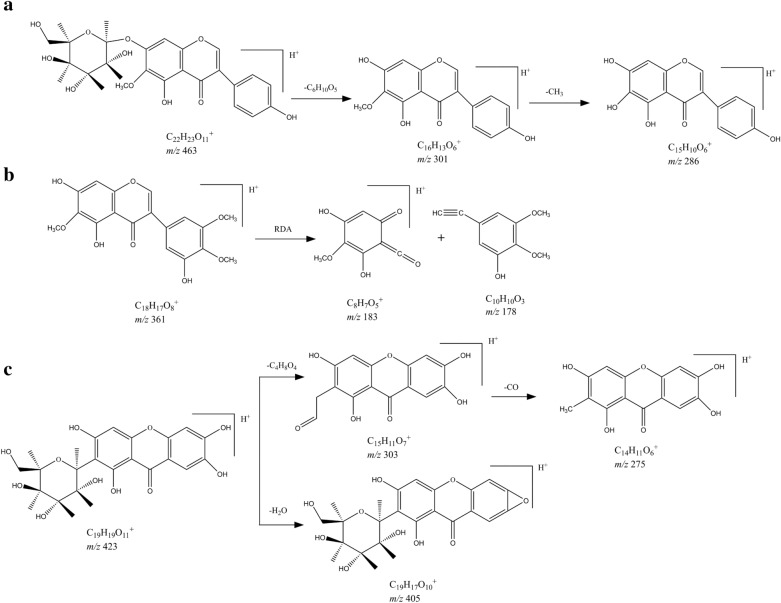
The proposed fragmentation pathways. **a** Tectoridin fragmentation, **b** Irigenin Retro–Diels–Alder (RDA) fragmentation (m/z 361→183), **c** Xanthone mangiferin fragmentation

## Data Availability

All data are fully available without restriction.

## Appendix

See Figs. 8 and 9.

## Abbreviations

BC: *Belamcanda chinensis* (L.) DC
ITM: *Iris tectorum* Maxim
DRS: Digital reference standard
RRT: Relative retention time
LCTRS: Linear calibration using two reference substances
TCM: Traditional Chinese medicine
UHPLC-HRMS/MS: Ultra-high-performance liquid chromatography-high resolution tandem mass spectrometry
Me-*β*-CD: Methyl-*β*-cyclodextrin
PCA: Principal components analysis
OPLS-DA: Orthogonal partial least squares-discriminant analysis
CCK-8: Cell counting kit-8

## References

[1] Woźniak D, Matkowski A (2015). Belamcandae chinensis rhizome—a review of phytochemistry and bioactivity. Fitoterapia.

[2] Chen Y, Wu CM, Dai RJ, Li L, Yu YH, Li Y, Meng WW, Zhang L, Zhang Y, Deng YL (2011). Combination of HPLC chromatogram and hypoglycemic effect identifies isoflavones as the principal active fraction of *Belamcanda chinensis* leaf extract in diabetes treatment. J Chromatogr B Analyt Technol Biomed Life Sci.

[3] Xie GY, Zhu Y, Shu P, Qin XY, Wu G, Wang Q, Qin MJ (2014). Phenolic metabolite profiles and antioxidants assay of three Iridaceae medicinal plants for traditional Chinese medicine “She-gan” by on-line HPLC-DAD coupled with chemiluminescence (CL) and ESI-Q-TOF-MS/MS. J Pharm Biomed Anal.

[4] Wu C, Li Y, Chen Y, Lao X, Sheng L, Dai R, Meng W, Deng Y (2011). Hypoglycemic effect of *Belamcanda chinensis* leaf extract in normal and STZ-induced diabetic rats and its potential active faction. Phytomedicine.

[5] Ito H, Onoue S, Yoshida T (2001). Isoflavonoids from *Belamcanda chinensis*. Chem Pharm Bull.

[6] Morrissey C, Bektic J, Spengler B, Galvin D, Christoffel V, Klocker H, Fitzpatrick JM, Watson RW (2004). Phytoestrogens derived from *Belamcanda chinensis* have an antiproliferative effect on prostate cancer cells in vitro. J Urol.

[7] Lee HU, Bae EA, Kim DH (2005). Hepatoprotective effect of tectoridin and tectorigenin on *tert*-butyl hyperoxide-induced liver injury. J Pharmacol Sci.

[8] Xiong Y, Yang Y, Yang J, Chai H, Li Y, Yang J, Jia Z, Wang Z (2010). Tectoridin, an isoflavone glycoside from the flower of *Pueraria lobata*, prevents acute ethanol-induced liver steatosis in mice. Toxicology.

[9] PerezdeSouza L, Alseekh S, Naake T, Fernie A (2019). Mass spectrometry-based untargeted plant metabolomics. Curr Protoc Plant Biol..

[10] Wang Q, Yu X, Sun L, Tian R, He H, Wang S, Ma S (2021). Fingerprint analysis of phenolic acid extract of Salvia miltiorrhiza by digital reference standard analyzer with one or two reference standards. Chin Med.

[11] Sun L, Jin HY, Tian RT, Wang MJ, Liu LN, Ye LP, Zuo TT, Ma SC (2017). A simple method for HPLC retention time prediction: linear calibration using two reference substances. Chin Med.

[12] Chen AZ, Sun L, Yuan H, Wu AY, Lu JG, Ma SC (2018). Simultaneous qualitative and quantitative analysis of 11 active compounds in rhubarb using two reference substances by UHPLC. J Sep Sci.

[13] Li J, Li WZ, Huang W, Cheung AW, Bi CW, Duan R, Guo AJ, Dong TT, Tsim KW (2009). Quality evaluation of *Rhizoma Belamcandae* (*Belamcanda chinensis* (L.) DC.) by using high-performance liquid chromatography coupled with diode array detector and mass spectrometry. J Chromatogr A.

[14] Chen YJ, Liang ZT, Zhu Y, Xie GY, Tian M, Zhao ZZ, Qin MJ (2014). Tissue-specific metabolites profiling and quantitative analyses of flavonoids in the rhizome of *Belamcanda chinensis* by combining laser-microdissection with UHPLC-Q/TOF-MS and UHPLC-QqQ-MS. Talanta.

[15] Wen Y, He L, Peng R, Lin Y, Zhao L, Li X, Ye L, Yang J (2018). A novel strategy to evaluate the quality of herbal products based on the chemical profiling, efficacy evaluation and pharmacokinetics. J Pharm Biomed Anal.

[16] Jiang H, Yue QI, Zou G, Guoxin LI (2017). Separation of isomer and similar structure compounds in Belamcandae Rhizoma by methyl-*β*-CD chiral mobile phase additive HPLC. Chin J Mod Appl Pharm.

[17] Forsberg EM, Huan T, Rinehart D, Benton HP, Warth B, Hilmers B, Siuzdak G (2018). Data processing, multi-omic pathway mapping, and metabolite activity analysis using XCMS online. Nat Protoc.

[18] Zhang YY, Wang Q, Qi LW, Qin XY, Qin MJ (2011). Characterization and determination of the major constituents in Belamcandae Rhizoma by HPLC-DAD-ESI-MS(n). J Pharm Biomed Anal.

[19] Wei Y, Shu P, Hong J, Qin M (2012). Qualitative and quantitative evaluation of phenolic compounds in Iris dichotoma Pall. Phytochem Anal.

[20] Ito H, Nishitani E, Konoshima T, Takasaki M, Kozuka M, Yoshida T (2000). Flavonoid and benzophenone glycosides from *Coleogyne ramosissima*. Phytochemistry.

[21] Ablajan K (2011). A study of characteristic fragmentation of isoflavonoids by using negative ion ESI-MSn. J Mass Spectrom.

[22] Liu S, Yan J, Xing J, Song F, Liu Z, Liu S (2012). Characterization of compounds and potential neuraminidase inhibitors from the *n*-butanol extract of compound Indigowoad Root Granule using ultrafiltration and liquid chromatography-tandem mass spectrometry. J Pharm Biomed Anal.

[23] Shu P, Hong JL, Gang WU, Yang YB, Qin MJ (2010). Analysis of flavonoids and phenolic acids in *Iris tectorum* by HPLC-DAD-ESI-MS~n. Chin J Nat Med.

[24] Duenas M, Hernandez T, Estrella I, Fernandez D (2010). Germination as a process to increase the polyphenol content and antioxidant activity of lupin seeds (*Lupinus angustifolius* L.). Food Chem.

[25] Kachlicki P, Einhorn J, Muth D, Kerhoas L, Stobiecki M (2008). Evaluation of glycosylation and malonylation patterns in flavonoid glycosides during LC/MS/MS metabolite profiling. J Mass Spectrom.

