# Supplementary material for: A novel multidimensional strategy to evaluate Belamcanda chinensis (L) DC and Iris tectorum Maxim based on plant metabolomics, digital reference standard analyzer and biological activities evaluation
Source: Chin Med. 2021 Aug 26;16:85. doi: 10.1186/s13020-021-00494-3 (PMC8393741; doi:10.1186/s13020-021-00494-3)
Supplement: Supplementary file 1 — Additional file 1: Table S1. Structures of all isoflavones found in Belamcanda chinensis (L) DC and Iris tectorum Maxim. Me: CH3, Glu: glucose. Table S2. Structures of isoflavones from Belamcanda chinensis (L) DC and Iris tectorum Maximwith additional dioxolane ring. Me: CH3, Glc: glucose. Table S3. Structures of xanthonesfrom Belamcanda chinensis (L) DC and Iris tectorum Maximwith. Me: CH3, Glu: glucose. Table S4. The absolute deviations (ΔtR) of the actual retention time and predicted retention time on nineteen columns in BC LCTRS method. Table S5. The absolute deviations (ΔtR) of the actual retention time and predicted retention time on nineteen columns in BC RRT method. Table S6. The absolute deviations (ΔtR) of the actual retention time and predicted retention time on nineteen columns in ITM LCTRS method. Table S7. The absolute deviations (ΔtR) of the actual retention time and predicted retention time on nineteen columns in ITM RRT method. Table S8. Neuroprotective and hepatoprotective effects of compounds and positive drug. [file 13020_2021_494_MOESM1_ESM.docx]

**Additional File 1 for**

A novel multidimensional strategy to evaluate *Belamcanda chinensis* (L) DC and *Iris tectorum* Maxim based on plant metabolomics, digital reference standard analyzer and biological activities evaluation

Hongxu Zhou^1,2#^, Yi Zhang^2#^, Hui Liang^1^, Huijie Song^1^, Jiaming Zhao^1^, Li Liu^1^, Jun Zeng^2^, Lei Sun^3^, Shuangcheng Ma^3*^, Dali Meng^1*^

^1^School of Traditional Chinese Materia Medica, Shenyang Pharmaceutical University, Shenyang 110016, PR China.

^2^Chongqing Institute for Food and Drug Control, Chongqing 401121, PR China.

^3^National Institutes for Food and Drug Control, Beijing 100050, PR China.

* Correspondence: masc@nifdc.org.cn, mengdali@syphu.edu.cn

^#^Both authors contributed equally.

Supplement Table 1 Structures of all isoflavones found in *Belamcanda chinensis* (L) DC and *Iris tectorum* Maxim. Me: CH_3_, Glu: glucose.

| Peaks | Compound | R1 | R2 | R3 | R4 | R5 | R6 | R7 |
| --- | --- | --- | --- | --- | --- | --- | --- | --- |
| P4/N9 | Tectorigenin-7-*O*-glucosyl-4'-*O*-glucoside | H | OMe | OGlc | H | H | OGlc | H |
| P5 | Tectorigenin-7-*O*-*β*-glucosyl (1-6) glucoside | H | OMe | OH | H | OH | OGlc-Glc | H |
| P6/N14 | Tectoridin* | H | OMe | OGlc | H | H | OH | H |
| P7/N19 | Iristectorin A* | H | OMe | OGlc | H | OH | OMe | H |
| P8 | 3'-Hydroxytectoridin | H | OMe | OGlc | H | OH | OH | H |
| P9/N20 | Iristectorin B* | H | OMe | OGlc | H | OMe | H | H |
| P10/N21 | Iridin* | H | OMe | OGlc | H | OH | OMe | OMe |
| P13/N26 | Tectorigenin* | H | OMe | OH | H | H | OH | H |
| P15/N29 | Iristectorigenin B* | H | OMe | OH | H | OH | OMe | H |
| P16/N30 | Iristectorigenin A* | H | OMe | OH | H | H | OMe | OH |
| P17/32 | Irigenin* | H | OMe | OH | H | OH | OMe | OMe |
| N11 | Iristectorigenin-A-7-*O*-*β*-glucosyl (1→6)-glucoside | H | OMe | OGlc-Glc | H | OH | OMe | H |
| N15 | Tectorigenin-4'-*O*-*β*-d-glucoside | H | OMe | OH | H | H | OGlu | H |
| N16 | Isotectorigenin-7-*O*-*β*-d-glucoside | H | H | OGlc | OMe | H | OH | H |
| N22 | Isoiridin | H | H | OGlc | OMe | OH | OMe | OMe |
| N24 | Iristectorin-B-4'-*O*-glucoside | H | OMe | OGlc | H | OMe | OGlc | H |
| N36 | Junipegenin C | H | OMe | OH | H | OMe | OMe | OMe |

Supplement Table 2 Structures of isoflavones from *Belamcanda chinensis* (L) DC and *Iris tectorum* Maximwith additional dioxolane ring. Me: CH_3_, Glc: glucose.

| Peaks | Compound | R1 | R2 | R3 | R4 |
| --- | --- | --- | --- | --- | --- |
| P12 | 3',5'-Dimethoxyirisolone-4'-*O*-*β*-D-glucoside | OH | OMe | OGlc | OMe |
| P18 | Noririsflorentin | OH | OMe | OMe | OMe |
| P19/N33 | Irilone | OH | H | OH | H |
| P20 | Irisflorentin* | OMe | OMe | OMe | OMe |
| P21/N37 | Dichotomitin* | OH | OH | OMe | OMe |
| N25 | Dichotomitin-3'-*O*-glucoside | OH | OGlc | OMe | OMe |

Supplement Table 3 Structures of xanthonesfrom *Belamcanda chinensis* (L) DC and *Iris tectorum* Maximwith. Me: CH_3_, Glu: glucose.

| Peaks | Compound | R1 | R2 | R3 | R4 | R5 | R6 |
| --- | --- | --- | --- | --- | --- | --- | --- |
| P1/N4 | Mangiferin | Glc | H | H | OH | OH | H |
| P2/N5 | Isomangiferin | H | Glc | H | OH | OH | H |
| P3/N6 | 7-*O*-Methylmangiferin | Glc | H | H | OH | OMe | H |
| N2 | Neomangiferin | Glc | H | H | OH | Glc | H |
| N8 | 7-*O*-Methylisomangiferin | H | Glc | H | OH | OMe | H |

Supplement Table 4 The absolute deviations (ΔtR) of the actual retention time and predicted retention time on nineteen columns in BC LCTRS method

| NO. | Tectoridin | Iristectorin A | Iristectorin B | Iridin | Tectorigenin | Iristectorigenin B | Iristectorigenin A | Irigenin | Irisflorentine | Dichotomitin |
| --- | --- | --- | --- | --- | --- | --- | --- | --- | --- | --- |
| t_R_ mean (min) | 27.51 | 29.44 | 31.87 | 32.90 | 47.33 | 53.85 | 55.38 | 58.90 | 72.57 | 73.98 |
| Col 1 | - | -0.0220 | 0.3067 | 0.0290 | 1.3090 | 0.1454 | 0.6534 | 0.3396 | 0.2360 | - |
| Col 2 | - | 0.1121 | -0.0552 | 0.0052 | 0.5197 | 1.1990 | 0.4232 | 0.5613 | 0.0486 | - |
| Col 3 | - | -0.0291 | 0.0444 | 0.1586 | 1.0610 | 0.9257 | 0.9893 | 0.6443 | 0.1200 | - |
| Col 4 | - | 0.1280 | 0.3368 | 0.3660 | 1.4290 | 1.4780 | 0.9702 | 0.7505 | 0.0735 | - |
| Col 5 | - | -0.0098 | 0.3031 | 0.2254 | 3.6940 | 1.9510 | 2.3800 | 2.0430 | 0.0557 | - |
| Col 6 | - | -0.0276 | -0.5001 | -0.4414 | -3.5150 | -3.1710 | -3.2140 | -1.7980 | -0.4937 | - |
| Col 7 | - | -0.0018 | -0.0519 | 0.0921 | -0.7732 | -0.4806 | -0.1470 | -0.5690 | -0.0421 | - |
| Col 8 | - | 0.1520 | 0.1633 | 0.2776 | 0.0554 | 0.9191 | 0.2685 | 0.1944 | 0.0813 | - |
| Col 9 | - | -0.1800 | -0.4377 | -0.5187 | -2.5760 | -2.006 | -1.4690 | -1.3460 | -0.0620 | - |
| Col 10 | - | -0.0258 | 0.0032 | 0.0484 | 0.3915 | 0.4501 | 0.5656 | 0.2001 | 0.0442 | - |
| Col 11 | - | 0.1114 | 0.2644 | 0.2439 | 4.19600 | 2.6410 | 2.1940 | 2.5330 | 0.1083 | - |
| Col 12 | - | 0.2121 | 0.5227 | 0.6034 | 3.8700 | 2.3100 | 2.3670 | 2.5010 | 0.0211 | - |
| Col 13 | - | 0.0760 | -0.1340 | -0.1292 | -0.6931 | 0.4030 | -0.2866 | -0.1689 | -0.0033 | - |
| Col 14 | - | -0.1487 | -0.6774 | -0.8133 | -3.7220 | -3.1850 | -3.1920 | -2.1120 | -0.4615 | - |
| Col 15 | - | -0.1806 | -0.5716 | -0.6429 | -3.2030 | -2.8280 | -2.0380 | -1.7940 | -0.1913 | - |
| Col 16 | - | 0.0021 | 0.1044 | 0.1250 | -1.8550 | -0.8620 | -0.8022 | -1.1400 | 0.0726 | - |
| Col 17 | - | -0.2099 | 0.2105 | 0.2151 | 0.1686 | 0.4435 | 0.3391 | -0.07268 | 0.1833 | - |
| Col 18 | - | 0.0091 | 0.0042 | -0.0342 | -0.9070 | 0.0585 | -0.2977 | -0.7432 | -0.0795 | - |
| Col 19 | - | 0.0370 | 0.2759 | 0.2959 | 1.2200 | -0.0370 | 0.7568 | 0.3031 | 0.3479 | - |
| 𝚫t_R_ mean (min) | - | 0.0882 | 0.2615 | 0.2771 | 1.8500 | 1.3420 | 1.2290 | 1.0430 | 0.1435 | - |
| SD | - | 0.1180 | 0.3377 | 0.3672 | 2.3740 | 1.7510 | 1.6220 | 1.3610 | 0.2064 | - |

Supplement Table 5 The absolute deviations (ΔtR) of the actual retention time and predicted retention time on nineteen columns in BC RRT method

| NO. | Tectoridin | Iristectorin A | Iristectorin B | Iridin | Tectorigenin | Iristectorigenin B | Iristectorigenin A | Irigenin | Irisflorentine | Dichotomitin |
| --- | --- | --- | --- | --- | --- | --- | --- | --- | --- | --- |
| t_R_ mean (min) | 27.51 | 29.44 | 31.87 | 32.90 | 47.33 | 53.85 | 55.38 | 58.90 | 72.57 | 73.98 |
| Col 1 | 1.7840 | 1.6340 | 1.7780 | 1.4500 | 1.6790 | 0.1568 | 0.5280 | - | -1.0110 | -1.3260 |
| Col 2 | -1.4010 | -1.2350 | -1.3430 | -1.2540 | -0.3465 | 0.5222 | -0.2340 | - | -0.1670 | -0.1799 |
| Col 3 | 0.1855 | 0.1072 | 0.1158 | 0.2002 | 0.7054 | 0.4049 | 0.4272 | - | -0.8648 | -1.0180 |
| Col 4 | 0.2443 | 0.3086 | 0.4357 | 0.4320 | 1.0150 | 0.8611 | 0.3217 | - | -1.0790 | -1.1940 |
| Col 5 | 1.2910 | 1.0890 | 1.1290 | 0.9557 | 2.6390 | 0.4206 | 0.6540 | - | -3.1520 | -3.3430 |
| Col 6 | -0.9634 | -0.8135 | -1.1030 | -0.9427 | -2.9560 | -1.9720 | -1.8760 | - | 2.7020 | 3.3730 |
| Col 7 | 0.4697 | 0.4741 | 0.4317 | 0.5794 | -0.2421 | 0.0724 | 0.4121 | - | 0.5727 | 0.6195 |
| Col 8 | -0.8052 | -0.6130 | -0.5544 | -0.4179 | -0.3653 | 0.6410 | 0.0075 | - | 0.1490 | 0.0934 |
| Col 9 | 0.8976 | -0.9469 | -1.0420 | -1.0520 | -2.1850 | -1.0870 | -0.3942 | - | 2.4010 | 2.5720 |
| Col 10 | 0.4499 | 0.3849 | 0.3632 | 0.3863 | 0.4254 | 0.3488 | 0.4303 | - | -0.4339 | -0.5062 |
| Col 11 | -0.5695 | -0.5762 | -0.5727 | -0.6519 | 2.2360 | 0.3944 | -0.1158 | - | -3.0750 | -3.2590 |
| Col 12 | 0.7908 | 0.7949 | 0.8392 | 0.8117 | 2.3590 | 0.3179 | 0.2210 | - | -3.5660 | -3.7220 |
| Col 13 | -1.5530 | -1.3660 | -1.4540 | -1.3920 | -1.1910 | 0.3249 | -0.3181 | - | 0.9283 | 1.0090 |
| Col 14 | -1.7420 | -1.6560 | -1.9340 | -1.9520 | -3.3440 | -1.8790 | -1.6840 | - | 3.6670 | 4.3750 |
| Col 15 | -0.9084 | -0.9293 | -1.1340 | -1.1180 | -2.5940 | -1.5900 | -0.5876 | - | 2.9970 | 3.3340 |
| Col 16 | 0.3177 | 0.3724 | 0.5434 | 0.5927 | -1.0480 | 0.1482 | 0.2512 | - | 1.6170 | 1.5810 |
| Col 17 | 1.1430 | 0.8741 | 1.1970 | 1.1660 | 0.6282 | 0.6711 | 0.5179 | - | -0.2198 | -0.4450 |
| Col 18 | -0.0143 | 0.0428 | 0.0978 | 0.0840 | -0.4537 | 0.6967 | 0.3697 | - | 1.0180 | 1.1340 |
| Col 19 | 1.6260 | 1.5430 | 1.6190 | 1.5750 | 1.5650 | -0.0120 | 0.6401 | - | -0.7898 | -1.2020 |
| 𝚫t_R_ mean (min) | 0.9028 | 0.8295 | 0.9309 | 0.8954 | 1.4720 | 0.6590 | 0.5258 | - | 1.6010 | 1.8050 |
| SD | 1.0790 | 0.9861 | 1.1030 | 1.0470 | 1.8140 | 0.8928 | 0.7184 | - | 2.0570 | 2.2980 |

Supplement Table 6 The absolute deviations (ΔtR) of the actual retention time and predicted retention time on nineteen columns in ITM LCTRS method

| NO. | Tectoridin | Iristectorin A | Iristectorin B | Iridin | Tectorigenin | Iristectorigenin B | Iristectorigenin A | Irigenin |
| --- | --- | --- | --- | --- | --- | --- | --- | --- |
| t_R_ mean (min) | 27.51 | 29.43 | 31.86 | 32.89 | 47.33 | 53.82 | 55.36 | 58.88 |
| Col 1 | - | -0.0795 | 0.2099 | -0.0832 | 1.0600 | -0.1248 | 0.3637 | - |
| Col 2 | - | 0.0699 | -0.1282 | -0.0903 | 0.1721 | 0.7169 | -0.0709 | - |
| Col 3 | - | -0.0201 | 0.0087 | 0.1338 | 0.3908 | 0.2448 | 0.3669 | - |
| Col 4 | - | 0.0869 | 0.2370 | 0.2333 | 0.9494 | 0.8303 | 0.3114 | - |
| Col 5 | - | -0.1359 | 0.0072 | -0.1317 | 2.2430 | 0.2476 | 0.5637 | - |
| Col 6 | - | 0.2176 | -0.0627 | 0.1103 | -1.8380 | -0.7885 | -0.6879 | - |
| Col 7 | - | 0.0315 | 0.0324 | 0.2064 | -0.3369 | 0.0485 | 0.4054 | - |
| Col 8 | - | 0.1166 | 0.1004 | 0.2253 | -0.0729 | 0.7439 | 0.1001 | - |
| Col 9 | - | -0.0725 | -0.1866 | -0.1954 | -1.6190 | -0.8598 | -0.2564 | - |
| Col 10 | - | -0.0752 | -0.0876 | -0.06292 | 0.1892 | 0.2642 | 0.3519 | - |
| Col 11 | - | -0.0454 | -0.0781 | -0.1769 | 2.4230 | 0.4842 | -0.0451 | - |
| Col 12 | - | 0.0451 | 0.1374 | 0.1197 | 2.1160 | 0.2115 | 0.1460 | - |
| Col 13 | - | 0.0933 | -0.1238 | -0.1157 | -0.6599 | 0.5000 | -0.1861 | - |
| Col 14 | - | 0.2380 | -0.0904 | -0.1534 | -1.8630 | -0.6606 | -0.5298 | - |
| Col 15 | - | -0.0717 | -0.3806 | -0.4206 | -2.2970 | -1.5070 | -0.5969 | - |
| Col 16 | - | 0.0825 | 0.2844 | 0.3502 | -1.1760 | 0.0951 | 0.2134 | - |
| Col 17 | - | -0.1809 | 0.2534 | 0.2670 | 0.2685 | 0.5212 | 0.3900 | - |
| Col 18 | - | 0.0492 | 0.1380 | 0.1140 | -0.3769 | 0.7497 | 0.4152 | - |
| Col 19 | - | 0.0111 | 0.2153 | 0.2261 | 1.0450 | -0.2713 | 0.4900 | - |
| 𝚫t_R_ mean (min) | - | 0.0728 | 0.1714 | 0.1977 | 1.1790 | 0.6106 | 0.4389 | - |
| SD | - | 0.0862 | 0.2090 | 0.2335 | 1.5140 | 0.8012 | 0.6152 | - |

Supplement Table 7 The absolute deviations (ΔtR) of the actual retention time and predicted retention time on nineteen columns in ITM RRT method

| NO. | Tectoridin | Iristectorin A | Iristectorin B | Iridin | Tectorigenin | Iristectorigenin B | Iristectorigenin A | Irigenin |
| --- | --- | --- | --- | --- | --- | --- | --- | --- |
| t_R_ mean (min) | 27.51 | 29.43 | 31.86 | 32.89 | 47.33 | 53.82 | 55.36 | 58.88 |
| Col 1 | 1.7330 | 1.5410 | 1.6630 | 1.3240 | 1.5100 | - | 0.3638 | -0.1941 |
| Col 2 | -1.5960 | -1.4610 | -1.5870 | -1.5140 | -0.7735 | - | -0.7633 | -0.5755 |
| Col 3 | -0.0751 | -0.1060 | -0.0911 | 0.0275 | 0.2017 | - | 0.1326 | -0.2521 |
| Col 4 | -0.1519 | -0.1152 | -0.0296 | -0.0590 | 0.2783 | - | -0.5445 | -0.9362 |
| Col 5 | 1.0410 | 0.8186 | 0.8368 | 0.6545 | 2.2140 | - | 0.2260 | -0.4814 |
| Col 6 | 0.0836 | 0.3194 | 0.1022 | 0.3106 | -1.2710 | - | 0.1487 | 2.1620 |
| Col 7 | 0.4246 | 0.4210 | 0.3783 | 0.5306 | -0.2621 | - | 0.3228 | -0.1386 |
| Col 8 | -1.0860 | -0.9432 | -0.9290 | -0.7894 | -0.9091 | - | -0.6324 | -0.6893 |
| Col 9 | -0.3913 | -0.3728 | -0.3731 | -0.3316 | -1.1150 | - | 0.7088 | 1.1510 |
| Col 10 | 0.3368 | 0.2199 | 0.1528 | 0.1536 | 0.0735 | - | 0.0509 | -0.3728 |
| Col 11 | -0.7792 | -0.8040 | -0.8102 | -0.8988 | 1.8890 | - | -0.5182 | -0.4337 |
| Col 12 | 0.5936 | 0.5790 | 0.5948 | 0.5462 | 2.0040 | - | -0.1103 | -0.3589 |
| Col 13 | -1.6620 | -1.4810 | -1.6030 | -1.5490 | -1.4910 | - | -0.6490 | -0.3021 |
| Col 14 | -0.9267 | -0.7570 | -0.9373 | -0.9320 | -1.7450 | - | 0.2486 | 2.1240 |
| Col 15 | -0.0400 | 0.0037 | -0.1729 | -0.1510 | -1.2440 | - | 1.0630 | 1.9170 |
| Col 16 | 0.2312 | 0.2889 | 0.4582 | 0.5105 | -1.1750 | - | 0.0978 | -0.1565 |
| Col 17 | 0.7766 | 0.5115 | 0.8073 | 0.7702 | 0.0734 | - | -0.1993 | -0.7410 |
| Col 18 | -0.3983 | -0.3746 | -0.3186 | -0.3556 | -1.0280 | - | -0.3501 | -0.8057 |
| Col 19 | 1.5990 | 1.5110 | 1.5810 | 1.5390 | 1.5800 | - | 0.6439 | -0.0008 |
| 𝚫t_R_ mean (min) | 0.7329 | 0.6647 | 0.7066 | 0.6815 | 1.0990 | - | 0.4092 | 0.7260 |
| SD | 0.9500 | 0.8523 | 0.9164 | 0.8614 | 1.3140 | - | 0.5049 | 0.9986 |

Supplement Table 8 Neuroprotective and hepatoprotective effects of compounds and positive drug.

| Concentration (μM) | Cell viability (100%) | | | | | | | | | |
| --- | --- | --- | --- | --- | --- | --- | --- | --- | --- | --- |
|  | Tectoridin | Iridin | Tectorigenin | Iristectorigenin B | Iristectorigenin A | Irigenin | Irisflorentine | Dichotomitin | Curcumin | Bicyclol |
| L02 (0.78) | 112.49±0.05 | 111.74±0.07 | 111.24±0.08 | 95.86±0.03 | 103.50±0.10 | 91.55±0.01 | 96.21±0.01 | 97.40±0.04 | - | 94.24±0.03 |
| L02 (1.57) | 127.05±0.11 | 123.40±0.16 | 116.12±0.02 | 91.52±0.09 | 101.00±0.06 | 89.55±0.03 | 102.87±0.08 | 92.53±0.05 | - | 97.82±0.07 |
| L02 (3.12) | 140.74±0.04 | 125.22±0.07 | 115.61±0.07 | 84.35±0.06 | 101.59±0.04 | 87.74±0.11 | 116.69±0.21 | 114.59±0.19 | - | 103.91±0.13 |
| L02 (6.25) | 141.71±0.08 | 133.15±0.12 | 113.82±0.09 | 93.77±0.08 | 102.87±0.05 | 91.08±0.09 | 113.63±0.19 | 108.55±0.08 | - | 99.19±0.07 |
| L02 (12.50) | 153.61±0.19 | 133.55±0.09 | 117.97±0.07 | 101.82±0.02 | 101.54±0.11 | 94.48±0.08 | 113.97±0.21 | 117.62±0.30 | - | 89.83±0.01 |
| L02 (25) | 145.10±0.18 | 127.04±0.20 | 92.89±0.10 | 104.72±0.30 | 90.89±0.07 | 83.01±0.21 | 103.10±0.24 | 103.25±0.12 | - | 79.73±0.06 |
| L02 (50) | 114.47±0.12 | 98.78±0.01 | 72.32±0.10 | 61.98±0.03 | 61.05±0.06 | 64.83±0.06 | 70.82±0.08 | 75.28±0.02 | - | 55.64±0.06 |
| L02 (100) | 63.22±0.09 | 48.30±0.01 | 47.44±0.03 | 40.53±0.02 | 27.58±0.03 | 34.81±0.01 | 36.05±0.02 | 32.53±0.04 | - | 29.01±0.04 |
| BV2 (0.78) | 115.10±0.07 | 120.93±0.03 | 106.71±0.03 | 80.20±0.01 | 100.83±0.10 | 84.95±0.01 | 85.58±0.02 | 81.29±0.09 | 108.26±0.13 | - |
| BV2 (1.57) | 103.38±0.07 | 105.71±0.14 | 106.12±0.02 | 72.70±0.01 | 98.40±0.06 | 67.81±0.01 | 69.61±0.02 | 70.50±0.02 | 104.79±0.11 | - |
| BV2 (3.12) | 97.53±0.04 | 97.66±0.06 | 102.48±0.04 | 68.20±0.04 | 98.97±0.04 | 68.87±0.02 | 75.12±0.06 | 77.87±0.02 | 106.46±0.05 | - |
| BV2 (6.25) | 106.65±0.06 | 109.12±0.08 | 109.63±0.04 | 64.59±0.06 | 100.22±0.06 | 62.28±0.08 | 67.82±0.04 | 69.18±0.01 | 99.03±0.10 | - |
| BV2 (12.50) | 95.28±0.03 | 105.72±0.10 | 112.12±0.11 | 63.72±0.04 | 98.92±0.04 | 56.44±0.01 | 65.08±0.05 | 61.37±0.07 | 106.58±0.10 | - |
| BV2 (25) | 92.58±0.11 | 107.37±0.09 | 96.12±0.08 | 52.11±0.02 | 88.55±0.02 | 64.41±0.03 | 55.03±0.03 | 61.68±0.01 | 75.21±0.13 | - |
| BV2 (50) | 88.12±0.07 | 109.26±0.07 | 94.94±0.12 | 48.08±0.01 | 59.47±0.01 | 57.13±0.01 | 52.64±0.01 | 57.10±0.01 | 14.38±0.02 | - |
| BV2 (100) | 65.58±0.03 | 79.93±0.03 | 53.01±0.02 | 43.01±0.01 | 26.87±0.01 | 32.69±0.01 | 30.25±0.01 | 39.29±0.04 | 15.15±0.01 | - |
| BRL-3A (0.78) | 95.59±0.14 | 92.72±0.04 | 93.66±0.06 | 99.14±0.12 | 91.03±0.19 | 98.84±0.04 | 91.20±0.29 | 99.13±0.04 | - | 89.93±0.01 |
| BRL-3A (1.57) | 87.48±0.09 | 84.56±0.03 | 86.07±0.03 | 99.05±0.08 | 79.71±0.13 | 93.63±0.13 | 91.76±0.02 | 97.43±0.07 | - | 86.09+0.02 |
| BRL-3A (3.12) | 93.07±0.21 | 91.65±0.03 | 90.15±0.05 | 100.49±0.03 | 93.38±0.07 | 95.43±0.12 | 93.21±0.07 | 98.06±0.07 | - | 86.49±0.05 |
| BRL-3A (6.25) | 88.31±0.04 | 90.12±0.15 | 92.91±0.03 | 96.13±0.13 | 91.69±0.04 | 90.19±0.09 | 87.46±0.08 | 94.81±0.09 | - | 88.97±0.03 |
| BRL-3A (12.5) | 90.91±0.04 | 96.40±0.19 | 94.53±0.02 | 102.85±0.06 | 92.57±0.07 | 94.21±0.07 | 96.07±0.08 | 87.62±0.44 | - | 92.77±0.03 |
| BRL-3A (25) | 93.03±0.11 | 94.37±0.05 | 92.89±0.01 | 99.54±0.04 | 92.34±0.05 | 95.75±0.10 | 88.01±0.10 | 80.92±0.05 | - | 85.54±0.06 |
| BRL-3A (50) | 91.51±0.14 | 94.92±0.05 | 93.65±0.02 | 95.21±0.07 | 89.76±0.06 | 89.52±0.09 | 74.88±0.09 | 67.48±0.09 | - | 66.05±0.05 |
| BRL-3A (100) | 77.49±0.13 | 79.77±0.14 | 72.67±0.05 | 59.71±0.12 | 51.40±0.08 | 49.90±0.08 | 43.51±0.13 | 43.50±0.13 | - | 27.58±0.04 |
